# Neurosensory development of the four brainstem-projecting sensory systems and their integration in the telencephalon

**DOI:** 10.3389/fncir.2022.913480

**Published:** 2022-09-23

**Authors:** Bernd Fritzsch, Karen L. Elliott, Ebenezer N. Yamoah

**Affiliations:** ^1^Department of Biology, The University of Iowa, Iowa City, IA, United States; ^2^Department of Otolaryngology, The University of Iowa, Iowa City, IA, United States; ^3^Department of Physiology and Cell Biology, School of Medicine, University of Nevada, Reno, Reno, NV, United States

**Keywords:** sensory map, sensory neurons, brainstem organization, midbrain, thalamus, telencephalon, multisensory integration

## Abstract

Somatosensory, taste, vestibular, and auditory information is first processed in the brainstem. From the brainstem, the respective information is relayed to specific regions within the cortex, where these inputs are further processed and integrated with other sensory systems to provide a comprehensive sensory experience. We provide the organization, genetics, and various neuronal connections of four sensory systems: trigeminal, taste, vestibular, and auditory systems. The development of trigeminal fibers is comparable to many sensory systems, for they project mostly contralaterally from the brainstem or spinal cord to the telencephalon. Taste bud information is primarily projected ipsilaterally through the thalamus to reach the insula. The vestibular fibers develop bilateral connections that eventually reach multiple areas of the cortex to provide a complex map. The auditory fibers project in a tonotopic contour to the auditory cortex. The spatial and tonotopic organization of trigeminal and auditory neuron projections are distinct from the taste and vestibular systems. The individual sensory projections within the cortex provide multi-sensory integration in the telencephalon that depends on context-dependent tertiary connections to integrate other cortical sensory systems across the four modalities.

## Introduction

Across vertebrates, the brainstem contains discrete nuclei which receive input from distinct peripheral sensory neurons. Neurons from these brainstem nuclei project to higher-order nuclei, permitting the relay of the specific sensory signal to unique areas of the telencephalon, where integration across sensory systems occurs. The telencephalon, or forebrain, is the last to form and develops as a bilateral expansion from a single end of the neuropore in the lancelet and the ascidians (Fritzsch and Martin, 2022). We will analyze four sensory systems (trigeminal, taste, vestibular, and auditory) from their peripheral nerve entry point within the hindbrain through their projection to the telencephalon. In addition, we will focus on the various genes expressed as well as the organizational principles for each system. For instance, while a topological organization exists for the somatosensory and the auditory projections to the telencephalon, the taste, and vestibular projections are not topologically organized. Moreover, higher-order interactions are provided to integrate the cohesive perception in the telencephalon that is documented chiefly in human organization.

Neurons of the peripheral nervous system (PNS) depend on specific genes for proper development (Kobayashi et al., 2019; Dvorakova et al., 2020; Xu et al., 2021). Most brainstem-projecting neurons differentiate by *Neurog2* expression from epibranchial placodes (Fode et al., 1998; Dennis et al., 2019), whereas all other remaining placodally derived neurons depend on *Neurog1* expression in mammals (Ma et al., 1998). There is a sequential expression of *Neurog1/Neurog2* in the spinal cord of the dorsal root ganglia (DRGs; Ma et al., 1999; Meltzer et al., 2021). DRGs in the spinal cord depend on *Sox10* followed by *Neurog2* for large and mediums sized neurons, and by *Neurog1* for small neurons and all depend on *Pou4f1* (Huang et al., 2001; Meltzer et al., 2021). In contrast, epibranchial and dorsal placodes develop independently of *Sox10* (Mao et al., 2013; Reiprich and Wegner, 2015). In mammals, the trigeminal, facial, glossopharyngeal, and vagus neurons depend on *Neurog1*, whereas the epibranchial neurons (geniculate, petrosal, and nodose) require *Neurog2*.

**FIGURE 1:**
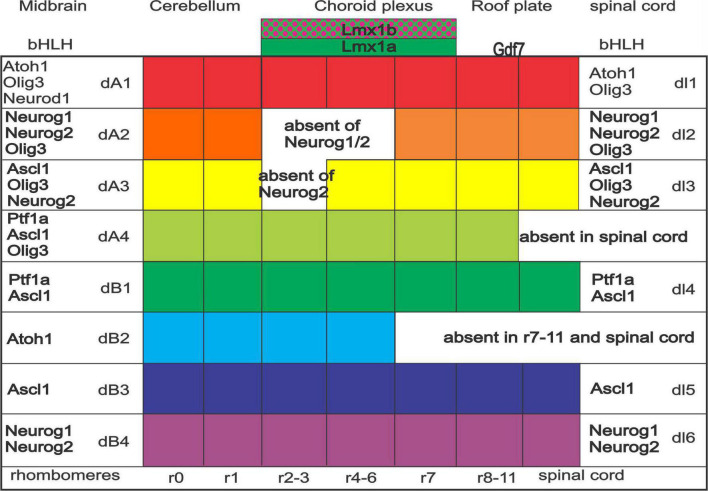
Expression of bHLH genes across spinal cord and brainstem. Specific and, in part, overlapping genes are expressed. Note that the spinal cord has only six (dl1-6) whereas the brainstem forms into eight expressions (dA1-4 and dB1-4). Particular expression is continuing, whereas specific gaps are unique among dA2 and dA3 (absence of *Neurog1* and or *Neurog2*), dA4 (*Ptf1a, Ascl1*, and *Olig3*), and dB2 (late expression of *Atoh1*). Rhombomeres are expressed in certain combined expressions (e.g., r2-3). Note that the choroid plexus depends on *Lmx1a/b* and *Gdf7*. Modified and combined after (Iskusnykh et al., 2016; Lai et al., 2016; Hernandez-Miranda et al., 2017; Chizhikov et al., 2021; Lowenstein et al., 2022).

The central nervous system (CNS) consists of the brainstem and spinal cord. In the brainstem, the dorsal alar plate is innervated by placodally derived peripheral sensory neurons, which depend on *Neurog1* or *Neurog2* (Fode et al., 1998). On the other hand, the spinal cord receives DRG projections which depend on *Neurog1/2* (Ma et al., 1999). These fibers extend ventrally to the basal plate in the spinal cord, including the muscle spindles (Lai et al., 2016; Meltzer et al., 2021). The CNS is divided dorsoventrally into the alar and basal plate, the former depending on choroid plexus formation along the dorsal part of the brainstem (Figure 1). Choroid plexus development requires *Lmx1a/b* expression, and without *Lmx1a/b*, neither the choroid plexus nor many of the dorsal alar plate structures form (Glover et al., 2018; Chizhikov et al., 2021). Downstream of *Lmx1a/b* is *Gdf7*, which is also necessary for roof plate formation (Lee et al., 2000; Mishima et al., 2009). The dorsoventral identity of neural progenitors within the developing dorsal brainstem and spinal cord are subdivided into eight or six distinct domains, respectively (Lai et al., 2016; Hernandez-Miranda et al., 2017; Hirsch et al., 2021). The most dorsal domain, dA1, is defined by an expression of *Atoh1* across the brainstem and spinal cord. Ventral to dA1 is the dA2 domain (*Neurog1/2* expression), followed by the dA3 domain (*Ascl1, Phox2b*, and *Tlx3* expression) rostrally, which forms the solitary tract (Qian et al., 2001). Caudally to the more rostral dA2/3, we have two neuronal populations of the brainstem: one is presented as a duplication of *Ptf1a* expression (dA4/dB1), whereas another set of unique populations from dB2 (*Atoh1, Phox2b*, and *Lbx1)* expression that forms, among others, the lateral vestibular nucleus (LVN) in rhombomere r4 (Chen et al., 2012; Lunde et al., 2019). While some of these domain populations in the hindbrain are maintained through the spinal cord, such as *Atoh1*, some gene expression differences lead to the absence of two domains in the spinal cord that are present in the brainstem (Hernandez-Miranda et al., 2017). Although *Atoh1* expression is continuous from the spinal cord to the cerebellum, separate nuclei are forming in this expression domain: the cerebellum (r0-1), the auditory nuclei (r2-5), and the pontine nuclei [r6-7 (Bermingham et al., 2001; Nichols and Bruce, 2006; Lowenstein et al., 2022)]. Further work is needed to detail the various nuclei and their specific migration to coalesce into distinct nuclei (r8-11; Rose et al., 2009; Watson et al., 2017a).

Second-order neuronal projections from the trigeminal, solitary tract, vestibular, and cochlear nuclei project in a relay to several additional nuclei before reaching the thalamus, whereas other second-order predictions are direct. Compared to the trigeminal projection that is common across vertebrates, information from taste, vestibular, and auditory nuclei is limited, and in addition, there is a unique loss of lateral line and electroreception in some species that are instead developing the auditory nuclei from frogs and amniotes (Grothe et al., 2004; Wullimann and Grothe, 2013; Fritzsch, 2021).

The bipartite of the telencephalon has long been seen as an exclusive input from the olfactory system of cyclostomes that is now shown to receive significant input from the trigeminal to the telencephalon in the lamprey (Suryanarayana et al., 2020, 2021), showing a basic telencephalon across vertebrates. Open questions concern the taste, vestibular, lateral line, electroreception, and auditory system in lampreys that show certain connections in gnathostomes (Striedter and Northcutt, 2019). It is unclear how the auditory system evolved beyond rudimentary insights that it is such a central system in mammals, frogs, reptiles, and birds (Briscoe and Ragsdale, 2019; Tosches and Laurent, 2019). Likewise, the taste system forms in all vertebrates but is unclear for higher-order connections to the telencephalon (Elliott et al., 2022a). The vestibular system has a basic understanding of the gnathostomes (Elliott et al., 2022a). Interestingly, there are multiple integrations of sensory systems. For example, auditory and visual input, taste and olfaction, and of vestibular and trigeminal (review in Shepherd, 2006; Majka et al., 2019; Chou et al., 2020; Mizoguchi et al., 2020; Rauschecker, 2021). We will provide the similarity of higher-order interactions of the four sensory systems and how they integrate multisensory modalities in the telencephalon with the four sensory systems.

## Structure of the sensory systems

*Somatosensory* receptors receive touch, proprioception, pain, and thermoreception, which project *via* trigeminal neurons to nuclei in the brainstem. Two major brainstem nuclei (principle and descending tract) send second-order projections mostly to the contralateral thalamus, and from there, the projections end up in the somatosensory topological cortex. Tertiary connections integrate other cortical sensory system inputs to direct motor output.

*Taste* buds, the peripheral end organs of gustation, contain chemosensory transducing cells. These cells are in the five different sensory taste buds innervated by four distinct sensory neurons. Three epibranchial placodes develop from *Neurog2*-expressing ectoderm to provide innervation from the oral cavity, particularly the tongue, and end in the solitary tract (ST) from r3-r11. From there, neurons project to the parabrachial nucleus (PB) to spread directly or indirectly in the thalamus and the insular cortex. Interactions of the olfactory system with vision, auditory, and vestibular provide an integrative taste perspective.

*Vestibular* hair cells and vestibular neurons detect and send, respectively, linear, and angular acceleration information to the brainstem. Various organizations and sensory epithelia are dedicated to detecting this directional information in gnathostomes. Vestibular neurons depend on *Neurog1* and provide innervation of hair cells at the periphery and end up in four distinct vestibular nuclei, as well as a part of the cerebellum (r1-r8) centrally. Within the cerebellum, vestibular neurons project a selected input to the uvula and nodulus. All spinal cord motoneurons receive an ipsilateral projection from the lateral vestibular nuclei (LVN), whereas three other vestibular neurons (superior, medial, and descending vestibular nuclei; SVN, MVN, and DVN) project to the three ocular motoneurons (III, IV, and VI). Bilateral outputs project the dorsal tegmental nucleus, the thalamus, and the parieto-insular vestibular cortex. Additional connections of auditory, visual, and taste information with vestibular input interact to drive higher-order spatial representations.

*Auditory* sensory neurons are unique neurons that evolved after the segregation of the basilar papilla developed. In mammals, auditory spiral ganglion neurons depend on *Neurog1* to innervate *Atoh1*-dependent hair cells. In mammals, three cochlear nuclei form only in r2-r5. Like the hair cells at the periphery, these cochlear nuclei depend upon *Atoh1* expression. Bilateral auditory nuclei interact with the superior olive nuclei (SOC) to go beyond the inferior colliculi (IC) to reach the medial geniculate body (MGB). The auditory cortex (aC) is the main target of the MGB, and auditory cortex neurons interact with those from vision, somatosensory, taste, and vestibular systems.

### Structure of the sensory systems and genes needed for their development

*Somatosensory sensory* inputs extend from the spinal cord to the trigeminal nuclei to provide somatosensory perception, giving rise to four brainstem PNS inputs (trigeminal, facial, glossopharyngeal, and vagal). These inputs reach rostrally from r2 and blend with the spinal cord past r11 caudally (Gray, 2013; Hans et al., 2020). The trigeminal nerve’s ophthalmic, maxillary, and mandibular branches project centrally in a ventral-to-dorsal orientation, ending with the small addition of other sensory neurons (Erzurumlu et al., 2010). The somatosensory input is comparable in the brainstem and the spinal cord: The spinal cord formed from *Neurog2* first to develop the largest and intermediate DRGs, followed by the population of *Neurog1*-dependent small DRGs (Ma et al., 1999; Meltzer et al., 2021). In contrast, brainstem-derived PNS neurons are exclusively developed from *Neurog1-*expressing cells (Ma et al., 1998) to innervate a mix of placode and neural crest (trigeminal) or neural crest-derived facial, glossopharyngeal (superior ganglion), and vagal sensory neurons (jugular ganglion). Most of the largest neurons reach the motoneurons in the spinal cord, except for the input from the trigeminal neurons.

A unique set of mesencephalic trigeminal neurons (MesV or MTN) develop that reach the brainstem and branch into a distal projection to innervate the muscle spindles of the trigeminal motoneurons (Fritzsch and Northcutt, 1993; Lipovsek et al., 2017; Ter-Avetisyan et al., 2018). The MesV develops in the absence of Neurog1 (Ma et al., 1998; Marzban et al., 2019), independent of the neural crest (Lipovsek et al., 2017). The largest proprioceptor neurons of the spinal cord depend on *Atoh1*, but these continuations are interrupted at the hindbrain to generate brainstem neurons giving rise to auditory and other neurons, the cuneate and gracile to continue the lateral lemniscus (Kuypers and Tuerk, 1964; Bermingham et al., 2001; Lai et al., 2016).

**FIGURE 2:**
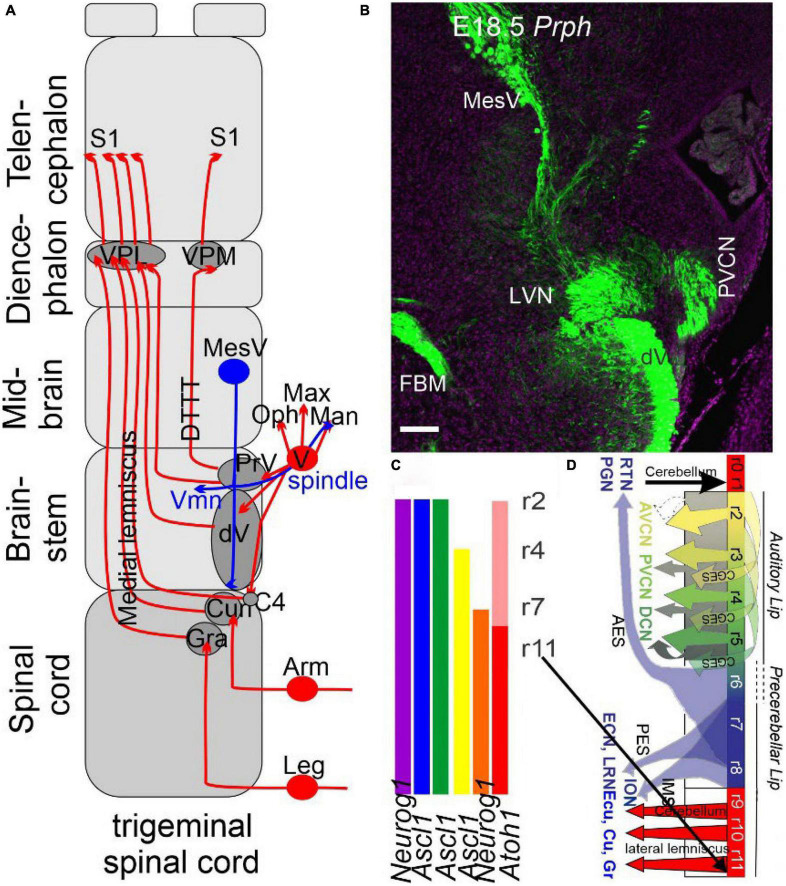
The trigeminal is projecting identical to the spinal cord. **(A)** Three trigeminal ganglia (ophthalmic, maxillary, and mandibular) project ipsi- and contralateral fibers from r2, whereas r3 of the principal trigeminal (PrV) and the descending trigeminal fibers (Vd) cross to the contralateral side, joining as medial lemniscus with the gracile (Gr) and cuneate (Cu) to reach the ventral posterior lateral nucleus (VPL) and ventral posterior medial nucleus (VPM) to reach the somatosensory cortex (S1). A unique projection is a mesencephalic tract (MesV, blue) to provide spindle fibers branching to the extent of the trigeminal motoneurons (Vmn) and the mandibular fibers. **(B)** A cross-section shows that all fibers are positive for peripherin (*Prph*), thus labeling the auditory, trigeminal, vestibular, facial, and MesV. **(C)** The organization of the primary central fibers is formed from *Atoh1, Neurog1*, and *Ascl1*, which have different central projections in the brainstem in dA1-4 and dB1-2. **(D)** Details of *Atoh1* show the formation of the granular neurons of the cerebellum (r0-1), cochlear (r2-5, AVCN, PVCN, and DCN), precerebellum (r6-8, PGN, and RTN) and reaches out the lemniscus fibers (r7-11) to become the gracile (Gr), cuneate (Cu), and the external cuneate (Ecu). The input from the inferior olive neurons (ION) is projecting to the contralateral cerebellum. AES, anterior extramural stream; CGES, cochlear granule cell extramural stream; Ecu, external cuneate; IES, intramural migratory stream; ION, inferior olivary nucleus; PES, posterior extramural stream; PGN, pontine gray nucleus; RTN, reticulotegmental nucleus. Modified after (Bermingham et al., 2001; Farago et al., 2006; Rose et al., 2009; Elliott et al., 2021a, 2022a).

Most *trigeminal* and proximal brainstem PNS neurons depend on *Eya1/2*, which is upstream of *Sox2* expression (Xu et al., 2021; Zhang et al., 2021). In addition, *Six1, Irx, Pax3, Dlx, Tbx*, and *Fox1* are expressed early. *Sox2* functions as a pioneer factor that regulates the expression of various bHLH genes; the trigeminal neurons depend on *Neurog1* expression (Ma et al., 1998; Kobayashi et al., 2019). Downstream of *Neurog1* are *Neurod1* and *Pou4f1*, which interact with *Isl1* to regulate all trigeminal ganglion neuron development. The first PNS neurons depend on *Tbx1/3*, which overlaps with the ophthalmic and maxillary ganglions. Further subdivisions are driven by *Oc2* (maxillary and mandibular ganglion) and *Oc1* and *Hmx2* (mandibular ganglion), forming discrete central projections to the CNS (Erzurumlu et al., 2010). An extensive set of distinct genes specify various sensory inputs and proteins fully described in the spinal cord input (Abraira and Ginty, 2013; Handler and Ginty, 2021) and the trigeminal ganglion as well as the MesV (Figures 2A–D). The expression of neurotrophin receptors differs between the spinal cord and trigeminal system for *TrkA, TrkB*, and *TrkC*. DRGs and trigeminal neurons depend on *Neurog1* and mostly *TrkA* (Ma et al., 1999). Loss of *TrkC* leads to the absence of DRGs innervating the muscle spindles, but since about 50% of muscle spindles form in the absence of *TrkC* suggests many MesV neurons remain (Matsuo et al., 1999; Huang and Reichardt, 2003). *Bdnf*, which preferentially binds to *TrkB*, reduces the number of MesV neurons when lost but seems not to affect the muscle spindles, whereas Merkel cells and hair innervation require *TrkC*. The interaction between *TrkC* and possibly *TrkB* remains unclear for the remaining trigeminal neurons. In summary, our data show a different dependence of trigeminal and spinal cord concerning *Neurog1/2* (spinal cord) compared to trigeminal, which depends on the placode and neural crest ganglions. A unique formation forms in the MesV that provide muscle spindle innervation but seems to develop independently of *Neurog1*, which diversifies into a set of neurons that depend on unique neurotrophins (Handler and Ginty, 2021; Meltzer et al., 2021).

*Taste* sensory information develops a unique step for taste buds in the oral cavity. Fungiform papillae depend on sonic hedgehog (*Shh*) and *Wnt* (Hall et al., 2003; Liu et al., 2007), while circumvallate papilla formations are regulated by *FGF10* and its receptors *Spry1-2* (Petersen et al., 2011). *Sox2* expression and innervation are required for continued taste bud development (Okubo et al., 2006; Fan et al., 2019). Taste buds are not differentiated when *Sox2* is conditionally deleted. The absence of *Neurog2* results in a reduction of *Sox2* expression (Okubo et al., 2006; Ohmoto et al., 2017) and causes taste bud formation to abort beyond a limited differentiation of small, single taste buds (Fan et al., 2019). Bitter-sensing taste buds seem to depend on *Eya1*, suggesting a unique expression of these sensory cells (Ohmoto et al., 2020). The differentiation of specific taste bud cells depends on numerous factors, only some of which have been identified (*Pou2f3, Ascl1*, and *Eya1)*.

**FIGURE 3:**
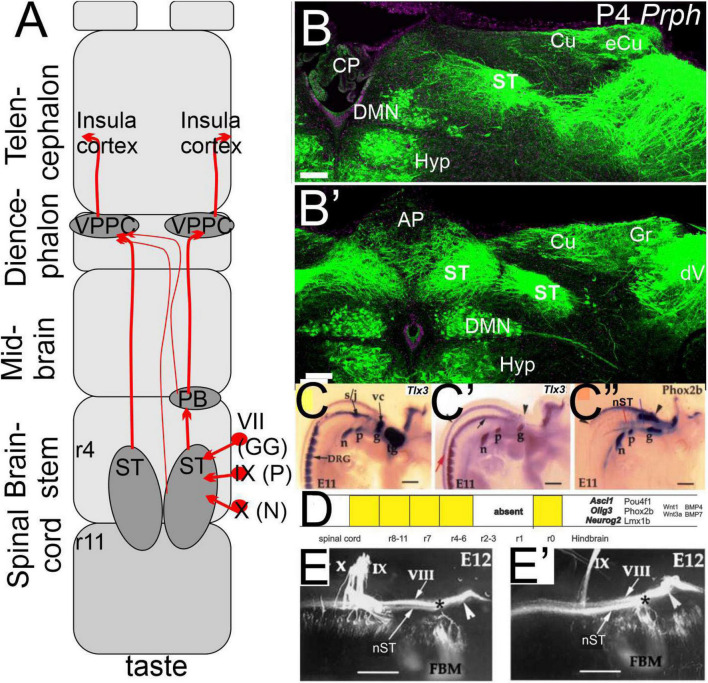
The taste buds are projecting *via* the solitary tract to innervate the insula cortex. **(A)** Three peripheral neurons (geniculate, petrosal, and nodose) project from r4-11 bilaterally to extend *via* the parabrachial nucleus (PB) and the thalamus (VPPC) to the insula cortex. **(B,B’)** sections show the solitary tract that is initially ventral to the vestibular nuclei that will replace more caudal to the external cuneate, cuneate, and gracile (eCu, Cu, and Gr). Note that the bilateral interaction is particularly large dorsal to the dorsal motor neurons (DMN) that interact with the area postrema (AP) that is replaced by the choroid plexus (CP). Central projection is positive for *Tlx3* **(C,C’)** and *Phox2b* **(C”)**, which define the dA3 **(D)**. Central projections show an identical pattern after the *Tlx3* is ablated **(E,E’)**. Modified after (Elliott et al., 2021a, 2022a; Roper et al., 2022). *VII entry point.

Neurons delaminate from the epithelium and migrate to achieve a final position near where three cranial nerves (facial, glossopharyngeal, and vagus) develop. Upstream of *Neurog2*, which defines the epibranchial placodes (Fode et al., 1998; O’Neill et al., 2012), is the expression of *Eya1, Sox2*, and *Pax2*. Downstream is the expression of *Neurod1, Isl1, Pou4f1, Phox2b*, and *Foxg1* (Alsina, 2020), which are needed to differentiate into the distal part of epibranchial neurons. *Phox2b* is explicitly required for a visceral sensory neuron fate (D’Autréaux et al., 2011), whereas other genes are needed for differentiation, such as a set of genes, *Coe1, Drg11*, and *Dcx.* This set of genes is only turned on after the migrating cells have left the placode (Freter et al., 2012; Smith et al., 2015). Following neuronal differentiation, developing gustatory neurons express *Ntrk2* (*TrkB*), required for the survival of all epibranchial neurons (Fritzsch et al., 1997). Ligands for the *TrkB* receptor, *BDNF* and *NT-4*, are expressed in the epibranchial neuron placodes and the taste buds (von Bartheld and Fritzsch, 2006; Huang and Krimm, 2010; Nosrat et al., 2012). Axons reach the correct target by making multiple decisions at several different locations along the path to the taste buds (Figures 3A–C). In summary, the development of both taste neurons and taste buds depends on a series of genes independent of one another: peripheral axons of neurons reach the taste epithelium. These two cell types become interdependent.

*Vestibular* neurons innervate vestibular hair cells located within the three canal cristae and two (mammals) or three (most gnathostomes; Fritzsch et al., 2002; Duncan and Cox, 2020) macula (Maklad and Fritzsch, 1999; Straka et al., 2014). Two vestibular neurons project to the vestibular hair cells (Desai et al., 2005): type I and type II, which innervate the two types of vestibular hair cells. The central projection of these neurons shows an incomplete and partial overlap of their target regions (Chagnaud et al., 2017; Elliott and Straka, 2022) that expands from the cerebellum rostrally to r8 caudally (Maklad and Fritzsch, 2003; Straka et al., 2014; Glover, 2020).

Vestibular neurons depend on certain common genes for specification and differentiation. For instance, no neurons develop without *Neurog1* (Ma et al., 2000). Upstream of *Neurog1* is the expression of the *Brg1*-based SWI/SNF chromatin-remodeling complex, which interacts with the neurosensory-specific transcriptional regulators of *Eya1/Six1* (Xu et al., 2021). These regulator genes induce the expression of *Sox2* (Dvorakova et al., 2020) to promote proneurosensory-lineage specification, such as upregulating *Neurog1* expression (Reiprich and Wegner, 2015; Kageyama et al., 2019). Downstream of *Neurog1* is *Neurod1*, which is important for neuronal differentiation; many neurons are lost without it. In addition, without *Neurod1* ectopic hair cells develop (Jahan et al., 2010; Macova et al., 2019). *Pou4f1, Isl1*, and *bHLHb5*, among others, are also needed for normal vestibular or auditory neuron development (Filova et al., 2020). The initial induction of an otic placode depends on *Foxi3* (Birol et al., 2016) and *Fg3/7/10* (Urness et al., 2011). Any differentiation of neurons or hair cells from this placode requires the expression of *Eya1* and *Pax2/8* (Zou et al., 2004; Bouchard et al., 2010). A disorganized development occurs following the loss of several genes, including *Lmx1a/b, Gata3, Dicer*, and *Shh*, among others (see reviews Riccomagno et al., 2002; Kersigo et al., 2011; Duncan and Fritzsch, 2013; Elliott and Gordy, 2020; Chizhikov et al., 2021). The loss of distinct neurons following specific gene deletion remains unclear (Diaz and Glover, 2021).

**FIGURE 4:**
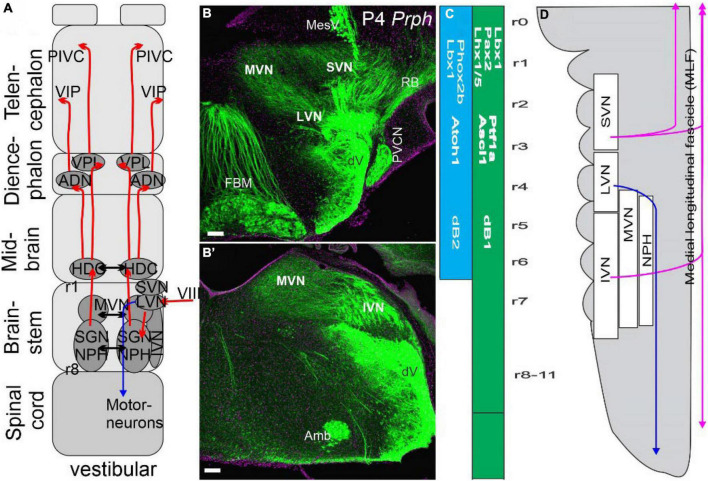
Vestibular neurons innervate to reach the cortex bilaterally *via* four nuclei. **(A)** Three vestibular nuclei send fibers bilaterally to the thalamus directly *via* the ventral posterior lateral nucleus (VPL) and indirectly *via* several steps to reach the anterior dorsal thalamus (AND). From there, projections extend to the distinct areas of the vestibular cortical area PIVC and the entorhinal cortex (VIP). Separate ipsilateral projections come from LVN to project to spinal motoneurons (in blue). **(B,B’)** A cross-section shows *Prph* expression in vestibular nuclei prominent LVN, SVN, and MVN that will continue as the IVN. **(C)** Two areas have been identified that form in dB1 and dB2. Note that dB2 is found between r1-r6 and is dependent on *Phox2b* and *Lbx1*. In contrast, dB1 continues with r0 to the spinal cord and is positive for *Ptf1a, Ascl1*, and *Lhx1/5*. **(D)** SVN, LVN, and IVN are ongoing, whereas the MVN is a separate, more medial organization next to the nucleus propositus hypoglossi (NPH). Note that ventral and dorsal fibers are indicated in color (blue for LVN, lilac for MLF). Modified after (Elliott et al., 2021a, 2022a; Elliott and Straka, 2022).

*Atoh1* is an essential gene for all mechanosensory hair cell development (Bermingham et al., 1999) beyond undifferentiated cells (Fritzsch et al., 2005). Vestibular hair cells differentiate into two types in amniotes: type I and type II (Elliott and Straka, 2022). The macula are divided into two distinct areas and are innervated by neurons that send central processes selectively to either the cerebellum or the brainstem, depending on the area of the macula that they innervate (Maklad and Fritzsch, 2003; Balmer and Trussell, 2019). *Emx2* is only expressed in one of these distinct areas (Holley et al., 2010; Jiang et al., 2017) and requires a detailed analysis of central connections to the cerebellum or the brainstem (Figures 4A–C). Hair cells within the utricle and saccule show a polarity that provides 360 degrees of sensitivity. In contrast, hair cells within a particular canal crista are all polarized in the same direction (Fritzsch et al., 2002; Elliott and Gordy, 2020). In summary, the vestibular neurons and hair cells require specific genes for proper development.

*Auditory* neurons innervate auditory hair cells located within the basilar papilla or cochlea. As with the vestibular system, there are two types of auditory neurons (type I and type II) that innervate two types of hair cells: inner hair cells (IHCs) and outer hair cells (OHCs) (Grabner and Moser, 2018; Jean et al., 2019; Moser, 2020). In mammals, these are arranged in a highly stereotyped organization with one row of IHCs and three rows of OHCs. The central projection of auditory neurons shows a highly organized tonotopic organization in auditory nuclei (Fritzsch et al., 2019).

As with vestibular neurons, auditory neurons also require the expression of *Neurog1* as these neurons never develop in the absence of its expression (Ma et al., 2000). *Neurog1* interacts with transcriptional regulators to promote the development of auditory neurons, the spiral ganglion neurons (SGN). In addition to *Neurog1*, these neurons depend on *Gata3, Pax2*, and *Lmx1a/b*: without any of these three transcription regulators, all SGNs are lost (Bouchard et al., 2010; Duncan and Fritzsch, 2013; Chizhikov et al., 2021). Downstream of *Neurog1* is *Neurod1* which regulates the differentiation of sensory neurons and their projections to the organ of Corti and the ventral cochlear nuclei (Jahan et al., 2010; Macova et al., 2019). In addition, SGNs require the expression of *Pou4f1, Isl1*, and *bHLHb5*, among others (Filova et al., 2020).

*Eya1*, followed by *Sox2*, is needed to initiate hair cell development (Dvorakova et al., 2020; Xu et al., 2021). As with vestibular hair cells, auditory hair cells also critically depend upon *Atoh1* expression (Bermingham et al., 1999). Downstream of *Atoh1*, hair cell differentiation requires *Pou4f1* and *Gfi1* (Xiang et al., 2003; Hertzano et al., 2004; Eng et al., 2007). Cochlear hair cells also need *Pax2, Gata3, Lmx1a/b*, and *Dicer:* without each case, there are no hair cells formed in the cochlear sac (Bouchard et al., 2010; Kersigo et al., 2011; Duncan and Fritzsch, 2013; Chizhikov et al., 2021). Unique genes are needed for the specific differentiation of IHCs (Nakano et al., 2020) and OHCs (Lorenzen et al., 2015; Chessum et al., 2018; Wiwatpanit et al., 2018). For instance, *Emx2* is needed to differentiate OHCs (Holley et al., 2010; Kindt et al., 2021). The total length of hair cells in the cochlea depends on *Neurog1, Neurod1, Foxg1, Prox1*, and *n-Myc* (Matei et al., 2005; Pauley et al., 2006; Fritzsch et al., 2010; Kopecky et al., 2011; Filova et al., 2020). Hair cells must be polarized with stereocilia aligned in a stair-step organization to function optimally. Specific genes are expressed that establish the distinct polarity of both vestibular and auditory hair cells (Montcouquiol et al., 2003). This polarity develops through steps, resulting in a kinocilium on one end (for vestibular hair cells; cochlear hair cells lack a kinocilium) connected with stereocilia that form a staircase (Fritzsch and Elliott, 2017; Elliott and Straka, 2022). After the polarity is established, *Pcdh15* and *Chd23* interact as tip links between taller and shorter stereocilia to respond to proper stimulation. Hair cells require a channel, *Tmc1/2*, to open, allowing an influx of K^+^ ions (Pan et al., 2013; Shibata et al., 2016; Erives and Fritzsch, 2020).

## Somatosensory—Developing distinct mechanosensory innervation

Nuclei dedicated for nociception (pain), thermosensation (temperature), pruriception (itch), mechanosensation (cutaneous/touch), and proprioception (limb and body position) are found in the brainstem and spinal cord (Erzurumlu et al., 2010; Lai et al., 2016; Meltzer et al., 2021). An extensive set of different neuronal categorizations is dependent on their size (largest and fast conducting Aα fibers, a variety of medium-sized conducting Aβ and Aδ fibers, and the thinnest unmyelinated C fibers) and provides proprioception (Ia, Ib, and II) that innervates the muscle spindle and tendon organs. The four mechanosensory cells are Merkel cells, Meissner, Ruffini, and Pacinian corpuscles (Abraira and Ginty, 2013; Handler and Ginty, 2021). Most of the innervation is driven by low-threshold mechanoreceptors (LTMR) for detecting mechanical stimuli, but there is also specific innervation for high-threshold mechanoreceptors (HTMR), which provide noxious input by thin, unmyelinated fibers (Meltzer et al., 2021).

Merkel cells depend on the local upregulation of *Atoh1* (Maricich et al., 2009) and *Pou4f3* for normal development. Like many other sensory innervations, these cells depend on *Piezo2* for mechanosensory cation channels, including the Aβ-LTMR innervation. Merkel cells are local next to the keratinocyte cells and show multiple indentations with desmosomes to interact with Merkel cells. An exciting feature is the presence of *Piezo2* in the afferent fiber and the Merkel cells, suggesting an interaction between the complex. Synapses form in Merkel cells, releasing them upon proper stimulations (Handler and Ginty, 2021). Compared to the *TrkC*-dependent Merkel cells that respond to forces of about 10 Hz, some fibers react to specific frequencies around 100 Hz (Meissner cells, which depend on *TrkB*). In comparison, Pacinian corpuscles are most sensitive to about 200–700 Hz and require *Ret* for proper innervation (Handler and Ginty, 2021). In summary, a wide variety of mechanosensory inputs depend on *Piezo2* to drive obviously in Merkel cells, a unique bHLH gene-dependent formation.

### Central nuclei differ between the brainstem and the spinal cord

A large set of expressed genes and proteins regulate the different central projections in the spinal cord (Lai et al., 2016; Handler and Ginty, 2021; Loutit et al., 2021) and the brainstem (Hernandez-Miranda et al., 2017; Hirsch et al., 2021). Whereas the spinal cord receives mechanosensory fibers from proprioceptors (muscle spindles), Aβ, Aδ, and C fibers, the trigeminal system in the hindbrain receives only Aβ, Aδ, and C from the trigeminal neurons to project the principle and the descending trigeminal branches that extend to C4. A separate projection from proprioceptors reaches the trigeminal motoneurons through bifurcations (Matsuo et al., 1999; Ter-Avetisyan et al., 2018) and provides information from the dental gums along the descending track (Lazarov, 2002). Trigeminal motoneurons are a part of the branchiomotor nuclei that belong to the facial, ambiguous, and dorsal motor nuclei (DMNs) that express *Phox2a/b* and *Lmx1b* (Figures 1, 2; Fritzsch et al., 2017). At least four dorsal nuclei receive trigeminal innervation, defined by the *Ascl1* gene (dA3, dA4, dB1, and dB3). In contrast, only three nuclei receive somatosensory information in the spinal cord (dl3-5; Figures 2A–D). A continuation of *Neurog1/2* (dl2) and *Atoh1* (dl1) defines the following expression in the spinal cord to regulate. Interestingly, this continued expression of *Atoh1* from the spinal cord to the cerebellum (dl1, dA1) shows a gap for *Neurog1* (dl2, dA2 to r7 and expression of r1) to add the cerebellum Purkinje cells (Hawkes, 2012; Obana et al., 2018). In the brainstem, the dorsal *Atoh1* expressing column gives rise to cerebellar granule cells (r1), the cochlear nuclei (r2-5), the pontine nuclei (r6-7), the pre-cerebellar nuclei (r7-8) and the external cuneate, the cuneate and the gracile (r9-11; review in Farago et al., 2006; Rose et al., 2009; Gray, 2013; Watson et al., 2017a; Arimura et al., 2019; Hirsch et al., 2021; Pop et al., 2021), that project to the cerebellum (pontine, external cuneate, inferior olivary nucleus; ION). Some areas expand from dA4 to expand from the ION, and depend on *Ptf1a* (Yamada et al., 2014; Iskusnykh et al., 2016; Hirsch et al., 2021), a unique contribution to the brainstem (Hernandez-Miranda et al., 2017).

Rodents best describe the detailed terminals of trigeminal neurons (Erzurumlu and Gaspar, 2020). Central projection of these neurons depends on *Hoxa2, Lmx1b, Epha4/7, Slit2, Robo1/2, Neuropilin1*, and *Sema3a* to define the maxillary branch that innervates the unique whiskers for normal central innervation of r2 (mandibular) and r3 (maxillary). Five rows of the maxillary projection were formed to develop a ‘barrelette.’ *Robo* deletions can cause a duplication from both ipsilateral and contralateral fibers to reach the somatosensory cortex (Iwasato and Erzurumlu, 2018). Retinoic acid (RA) influences the patterning of *Hoxa2* (Glover et al., 2006). *Lmx1b* acts upstream of *Fgf8* and limits the rostral distribution of *Hoxa2* to r2 (Glover et al., 2018). Fate mapping shows the principal trigeminal nucleus (PrV) is derived from r2 and r3 and the spinal cord (caudalis; SpV) to present an inverted projection that starts in the ophthalmic region to the more dorsal mandibular fibers in a topographical projection of the face. Lower jaws and lips project from the mandibular ganglia to r2, whereas the maxillary fibers project to r3 to innervate the ‘barrelette’ (Erzurumlu et al., 2010). Second order trigeminal neurons give rise to the contralateral lemniscal of SpV, whereas the PrV projects to the thalamus to innervate the VPM (Erzurumlu et al., 2010; Iwasato and Erzurumlu, 2018). Mutants have disorganized central projections that lack the ‘barrelette’ formation (*Drg11, Nmdar1, and Lmx1b*; reviewed in Erzurumlu and Gaspar, 2020).

In humans, we can define between the PrV, which forms a dorsomedial division, and a ventrolateral division, likely belonging to r2 and r3, respectively. The dorsomedial provides the dorsal trigeminothalamic tract (DTTT; Figure 1A) to reach the ipsilateral VPM, whereas the ventrolateral division, the ventral and dorsal trigeminothalamic tracts (VTTT, DTTT) that crosses to innervate, like other trigeminal lemniscus fibers, the VPM next to the VPL in humans (Suer, 2021) and monkeys (Andrew et al., 2020). It requires a detailed analysis in mice for the ipsilateral projections from r2. A discrete projection from the PrV projects predominantly ipsilateral fibers. *Atoh1* is expressed from the cerebellum to the spinal cord but shows a regular formation to generate granular cells, auditory nuclei, pontine, and ION, and receives the external cuneate, cuneate and gracile between r1-r11 (Figure 2). At least four *Ascl1* positive genes (dA3, dA4, dB1, and dB3) are driving, in part, the development of trigeminal nuclei. In summary, the spinal and trigeminal central projections are contralateral and are, in part, like the medial lemniscus in the SpV, cuneate, and gracile.

### The thalamus receives the topological somatosensory inputs

**FIGURE 5:**
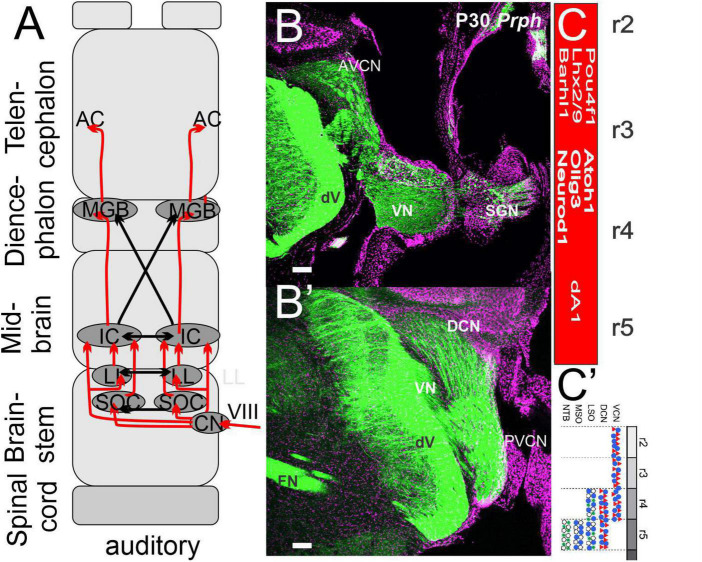
Cochlear neurons innervate three nuclei forming bilaterally in the auditory cortex. **(A)** Three cochlear nuclei (AVCN, PVCN, and DCN) project bilaterally to an intermediate set of SOCs and LL to innervate directly or indirectly the IC. Crossing fibers are prominent that project to the auditory cortex (A1). **(B,B’)** Sections reach different areas, shown with *Prph*. **(C,C’)** A single cochlear nuclei depend on *Atoh1, Olig3*, and *Neurod1* giving rise to the SOC from r4/5. Modified after (Lipovsek and Wingate, 2018; Elliott et al., 2021a, 2022a).

In mice, fibers reach the VPM to innervate as ‘barreloids’ the thalamic nucleus (Erzurumlu et al., 2010) from r3 projections, while the spinal fibers enter as the contralateral medial lemniscus (SpV, gracile, and cuneate) to innervate the VPL (Iwasato and Erzurumlu, 2018). Interesting bilateral information is described in gums and teeth that show faster ipsilateral projections in humans and monkeys (Andrew et al., 2020; Hihara et al., 2020). We have a somatosensory, topological innervation (Figures 1A, 5A) to project to the mammalian cortex with a primary and secondary innervation (Liao et al., 2020; Qi et al., 2020; Zilles and Palomero-Gallagher, 2021). Based on the earliest description of Brodmann’s work, they defined cortical organizations that were further distinct, showing the ‘homunculus’ by Penfield. In addition, lampreys and gnathostomes show a similar direct trigeminal projection that reaches, with VPM/VPL, the cortex (Briscoe and Ragsdale, 2019; Suryanarayana et al., 2020).

Mice, and other rodents, have a unique organization, the barrel field, a significant innervation that can be manipulated by loss and gain of input (Erzurumlu and Gaspar, 2020). The cortical organization is affected by changing the pattern of central projections. For example, sensory receptors of whiskers differentiate after birth and can change the pattern from the brainstem (barrelettes), thalamus (barreloids), and the cortex (barrel field). Further, manipulations have studied the effect of plasticity on morphological and physiological plasticity. *Fgf8* defines the rostromedial neocortical primordia that organize somatosensory cortical maps. Calcium signal drives the thalamus and barrel field before sensory input is present, and without calcium, it results in hyperexcitability to provide projections to the barrel field organization (Antón-Bolaños et al., 2019). The organization is controlled by specific transcription factors with dendrites that suggest the self-organization of a proto-map assembly to drive thalamocortical sensory circuits (Steiner et al., 2020). Cross-modal plasticity can substitute input modality during development, which has important implications for plasticity and evolution after surgical replacement of part of the cortex (Pallas, 2001; Kral and Pallas, 2011). In summary, the developing somatosensory input generates the topological input that all mammals and other vertebrates provide. The role of innervation depends on specific inputs to create the barrel field in mice and can be used as cortical reorganizations.

## Taste buds innervate four sensory neurons from five-sensory receptors

Four types of taste bud cells have 30 to 150 tall columnar cells in the orofacial innervation. The largest population (about 50% of cells) are *Type I* cells, glia-like cells. *Type II* and *Type III* cells are receptor cells for the 5 basic taste qualities (sweet, salty, sour, bitter, and umami). *Type IV* cells are considered immature cells that do not extend to the taste and differentiate into other cell types over the next 20 days (Perea-Martinez et al., 2013; Ohmoto et al., 2020). *Type II, III*, and *IV* contribute to 19, 15, and 14% of taste bud cells, respectively (Yang et al., 2020).

*Type II* taste cells are chemosensors that express G protein-coupled taste receptors (*Tas1Rs, Tas2Rs*) to detect sugars, amino acids, and bitter compounds. *Sugars* bind to the dimeric receptor *Tas1R2/Tas1R3* while *umami* activates the dimeric receptor *Tas1R1/Tas1R3.* It is unknown whether *Tas2Rs* form dimers in *bitterness* (Kinnamon and Finger, 2019; Ohmoto et al., 2020; Roper, 2020). *Salt/NaCl* taste responds to a subset of Type II cells to release neurotransmitters (Bigiani, 2021). *Type III* taste cells form conventional synapses with sensory afferent fibers and respond to *sour* tastes (Roper, 2020). These cells respond to a combination of (1) intracellular acidification and (2) proton (H^+^) influx through apical OTOP1 channels (Teng et al., 2019; Zhang Y. et al., 2020).

Six neurotransmitters have been identified in taste buds: ATP, 5-HT, GABA, ACh, glutamate, and noradrenaline (see review Roper and Chaudhari, 2017; Roper, 2021), with receptors expressed on the postsynaptic sensory afferent terminals. Individual taste cells and gustatory afferent nerve fibers respond either to single tastes (sweet, salty, sour, and umami) or, more broadly, to several different taste qualities (Wooding and Ramirez, 2020) that are more compatible with cross-fiber or combined tastes (Roper, 2020). A subset of neurons innervates taste buds that express the mechanosensitive channel *Piezo1*, which may play a role in the tactile responsiveness of taste buds (Dvoryanchikov et al., 2017). In summary, three sensory cells (sweet, bitter, and umami) are sensory *Type II* cells, whereas sour cells are *Type III* cells that synapse onto neurons. Salt sensation is unclear to date.

### The solitary tract depends primarily on three genes

Central projections of three different nerves for taste perception (geniculate, nodose, and petrosal) show initially entirely overlapping terminal fields (Figures 3A–E); each nerve occupies a discrete, overlapping territory, suggesting a weak oral topography (Hill and May, 2007; Lundy and Norgren, 2015). A ‘homotopy’ is more pronounced in lampreys and lower gnathostomes than in mammals (Finger, 1978; Fritzsch and Northcutt, 1993; Daghfous et al., 2020). It raises the question of the functionality of constant input (Finger, 2008) and suggests further investigation (Lundy and Norgren, 2015).

Centrally, taste information ends up in the nucleus of the solitary tract (ST or NST; Rose et al., 2009; Gray, 2013; Hernandez-Miranda et al., 2017). The development of the ST depends on a combination of bHLH genes (*Ascl1, Neurog2*, and *Olig3)* that outline the dA3 brainstem through the spinal cord (Figures 3A,B). The genes, *Tlx3, Lmx1b*, and *Phox2b* are co-expressed in the neurons of the ST but show a different longitudinal expression that is prominent for *Phox2b* in the dA3 (r1-r11) that end before the DRGs (Qian et al., 2001; Hirsch et al., 2021). *Phox2b* is essential for the viscerosensory fate that determines the dA3 that convert into dB3-like development (D’Autréaux et al., 2011). In contrast, *Tlx3* expressing neurons project from the cerebellum and continue from r4 through the spinal cord (Figure 3; Qian et al., 2001). *Lmx1b* is expressed throughout the cerebellum and spinal cord (Mishima et al., 2009). A fusion of the left and right ST forms a prominent input that reaches r8-11 and forms the area postrema that expands ventrally to the gracile and overlaps with the hyoglossus (XII; Gray, 2013).

A subpopulation, which expresses the dA3 markers *Phox2b/Lmx1b* (Figures 3C–E), is localized to the ST by projecting to the parabrachial nucleus (PB) and reaching out to the pre-locus coeruleus (pLC) complex in the prepontine hindbrain (r0-1; Watson et al., 2017b; Gasparini et al., 2021) to subdivide PB as depending on *Lmx1b* and *Phox2b*. A large regulation provides homeostatic functions, hunger, thirst, sodium appetite, taste aversion, and cardiorespiratory influence (Karthik et al., 2022). Other ST reaches ventrally and caudally to reach different cervical and thoracic spinal cord segments to innervate pre-motor neurons (Hirsch et al., 2021), among many other connections (Lundy and Norgren, 2015). In summary, taste buds send taste information through three cranial nerves (VII, IX, and X) to the hindbrain that develops specific gene expression (dA3) to innervate the solitary tract (r4-11) that retains a rough orthotopic organization.

### Thalamus provides the input to the insula

Solitary tract neurons send output projections to a variety of different targets, including the PB as one of the heaviest targets to receive input from the isthmus (r0) and rhombomere 1 (r1; Watson et al., 2017b). The PB neurons relay *via* gustatory and general visceral input to the “gustatory/visceral” thalamic region that also receives input from the ST directly (Figure 3A). The thalamic gustatory relay is referred to as ventral posterior thalamic nucleus parvocellular (VPPC) and is defined as a ‘taste relay’ (Herbert et al., 1990; Lundy and Norgren, 2015). This unique, predominantly ipsilateral connection breaks down into a complex interaction from the VPPC that receives fibers from the ipsi- and, to a lesser extent, the contralateral PB. The largest PB injections reach the medial septum, olfactory tubercle, and receive the gustatory cortex (Saper and Loewy, 1980; Lundy and Norgren, 2015). The orthotopic organization in the ST is not preserved in higher-order nuclei, making the need for any orthotopic primary map even fuzzier (Lundy and Norgren, 2015). PB neurons project axons indirectly to the insular cortex *via* VPPC (reviewed in Lundy and Norgren, 2015; Gasparini et al., 2021).

In primates, including humans, the central gustatory pathway bypasses the PB, projects directly to the VPPC, and reaches the granular region of the insular cortex (Lorenzo, 2021). Highly conserved second-order neurons project taste information to the cortex (Lundy and Norgren, 2015; Vendrell-Llopis and Yaksi, 2015; Staszko and Boughter, 2020). The major terminal field in the insula region interacts with other inputs, in addition to taste (Fain, 2019; Staszko et al., 2020). In contrast to the clear-cut neuroanatomical pathways for taste, a uniform interpretation of taste coding has yet to emerge in the insula cortex. Imaging studies have conflicting reports (Chen et al., 2011, 2021), and many studies report a broad distribution of taste neurons throughout the insula cortex with no spatial organization (Staszko et al., 2020), including fMRI studies in human subjects (Canna et al., 2019; Avery et al., 2020). Gustatory responses were elicited from the anterior tongue and posterior tongue, and pharyngeal responses were located caudal to the anterior tongue (Hanamori et al., 1998). The reciprocal connection between the cortex, PB, and ST indicates descending projection from forebrain areas converging the brainstem gustatory neurons, the PB, and the ST into an integrated interaction. In summary (Figure 3A), gustatory PB neurons reach the taste cortex without or with a relay with the thalamus and have reciprocal connections with the gustatory and visceral cortex directly from the PB.

## Vestibular nuclei develop from two areas in the hindbrain

Vestibular neurons innervate the semicircular canals, and otolithic organs at the periphery and project centrally to the vestibular nuclei (Straka et al., 2014). Mammals have four defined nuclei: the descending, lateral, medial, and superior vestibular nuclei (DVN, LVN, MVN, and SVN). Nuclei that form the SVN are from r1-3, LVN mostly from r4, DVN from r5-8, and MVN from r2-8 (Chagnaud et al., 2017; Glover, 2020; Elliott and Straka, 2022). Vestibular afferents project partially overlapping and separate within the vestibular nuclei (Figures 3A–D). There is no topologically organized central or peripheral organization compared to the trigeminal system, which is more akin to the taste input (Fritzsch et al., 2019). Within the overlap of different central projections, one can distinguish them into incomplete projections in partial central information. For example, the cerebellum receives only fibers from the saccule to the uvula, whereas the posterior canal is prominent to the nodulus and has a limited expansion to the uvula (Maklad and Fritzsch, 2003; Balmer and Trussell, 2019), like the remaining vestibular organization (Elliott and Straka, 2022). Central projections depend on the expression of specific genes. For example, in the absence of *Neurod1, Gata3*, or *Fzd3*, there is an overlap of cochlear and vestibular central projections (reviewed in Duncan and Fritzsch, 2013; Duncan et al., 2019; Filova et al., 2020; Stoner et al., 2021). *Lmx1a/b* double null mice cross afferents to the contralateral side after the choroid plexus is eliminated (Chizhikov et al., 2021).

Vestibular nuclei develop from at least two longitudinal central nuclei progenitor populations: dB1 and dB2 (Hernandez-Miranda et al., 2017; Hirsch et al., 2021). dB1 expresses *Ascl1* and *Ptf1* from the spinal cord to the cerebellum (r0-r11, spinal cord). Additional genes expressed are *Lbx1, Pax2*, and *Lhx1/5* (Diaz and Glover, 2021). In contrast, a unique gene expression is present in dB2, *Phox2b*, and *Lbx1*, from r1 to r6. A later manifestation of *Atoh1* is reported (Hirsch et al., 2021). Gene expression of *Hoxb1* overlaps with r4 and depends on *Phox2b* and *Lbx1* that define the LVN, reaching the ipsilateral spinal motor neurons (Chen et al., 2012). Further, developing mice (E13-15; Lunde et al., 2019) show a nearly equal expression of *Esrrg, Maf*, and *Pou3f1* in LVN/MVN. In contrast, *Lhx1/5, Evx2*, and *Foxp2* are expressed in the nearby MVN in r5 that project in a different direction with additional expression of several other genes (Lunde et al., 2019). In addition, a reduced expression level in *Onecut1-3* is higher in the MVN compared to the LVN (Glover, 2020). Additional expression is possible from other origins (Figures 4A,D). In particular, dB4 is positive for bHLH genes *Neurog1* and *Neurog2* (Diaz and Glover, 2021). Gene expression changes by migration later, following in part the information from neurons developing spinal and brainstem neurons (Lai et al., 2016; Hernandez-Miranda et al., 2017). Further work is needed to define the contribution of at least two earliest gene expressions (dB1, dB2) that originate along the longitudinal gene expression from r1-r8 (Diaz and Glover, 2021; Hirsch et al., 2021). In summary, at least two areas are defined to add distinct vestibular nuclei (Figures 3A,C,D) that indicate other origins, making it a complex migration from different neuronal populations (reviewed in Diaz and Glover, 2021).

### Neurons from the thalamus project to the insula to provide vestibular information

Semicircular canal organs converge with otolithic organs to generate an end-organ-specific central organization that is incompletely presented in overlapping and distinct functional inputs (Straka et al., 2014; Elliott and Straka, 2022). Considerable overlap of vestibular end-organs precludes defining a classical sensory map as the organizational principle (Chagnaud et al., 2017). Three major 2^o^ order output fibers are determined from the vestibular nuclei:

1. The LVN gives rise to the ipsilateral projection to the spinal cord motoneurons *via* the lateral vestibulospinal tract (LVST).
2. The MVN, SVN, and DVN project bilaterally to the ocular motoneurons (III, IV, and VI).
3. Additional fibers project to the lateral mammillary and dorsal tegmental nucleus to extend *via* the anterodorsal and ventral posterior lateral thalamus (AND, VPL) to the parietal-insula vestibular (PIVC) and the ventral intraparietal cortex (VIP).

We concentrate on the thalamocortical interaction (Figure 4A), providing a limited discussion on the two motor outputs of the LVN to the spinal cord, as all other vestibular fibers project to the ocular motoneurons (see review Fritzsch et al., 2017; Glover, 2020; Elliott and Straka, 2022). Within this connection is the cerebellum that receives and emits fine control of movements and storage of velocity and activates visual-vestibular integration using the nucleus propositus hypoglossal and contacts with the parabrachial nucleus to deal with cardiovascular and respiratory control (Cullen et al., 2021; Dieterich and Brandt, 2021; Elliott and Straka, 2022). The vestibular-ocular reflex (VOR) provides active and passive head-body motion, whereas the optokinetic reflex (OKN) provides visuomotor responses (Figure 3A).

Vestibular projections are bilateral, primarily from the vestibular nuclei to the thalamus and cortex. Certain areas may project ipsilaterally and contralaterally to the cortex. It appears that certain stimuli project *via* ipsilateral connections to the extent of the PIVC in humans. However, other stimuli project bilaterally or even a dominant contralateral input that reaches across three different levels to innervate the cortex (Dieterich et al., 2017), showing an asymmetric interaction from vestibular nuclei (VN) followed by a dominant contralateral input that crosses back except for the thalamus (Dieterich and Brandt, 2018). At least three midline crossings are located: (1) at the level of the vestibular nuclei; (2) at the pontine level above the vestibular nuclei; (3) at the mesencephalic level (Dieterich and Brandt, 2021). Vestibular nuclei project *via* a relay to pass through the nucleus propositus hypoglossal (NPH) and reach the supragenual nucleus (SGNu) that plays a role in the head direction network (HDC; Cullen et al., 2021; Taube and Yoder, 2021). From here, projections expand to the anterior vestibular-thalamic pathway (ADN) in the thalamus, and, from there, it projects to the limbic and entorhinal cortex. In addition, vestibular projections project bilaterally to the ventral posterolateral nucleus (VPL) that innervates the parietal insula of the vestibular cortical area (PIVC). In addition to the parietal-insula vestibular area (PIVC), vital information is sent to the visual temporal Sylvian area (VTS) in the retroinsular cortex, the superior temporal gyrus (STG), the inferior parietal lobule (IPL), the anterior cingulum, the hippocampus, and area 6a. Posterolateral thalamic nuclei project bilaterally *via* VOPL, VPM, and VL (Dieterich et al., 2017) to end up in the PIVC, but some fibers from the brainstem can project directly to the PIVC. Overall, fiber tracking data in humans shows that both the VPL (crossed and uncrossed projections) and VP (uncrossed projections) thalamus serve as vestibular relay stations for brainstem projections to (or from) the vestibular cortex.

Moreover, the ventral intraparietal areas (VIP) play a role in the vestibular circuits, where they interact with somatosensory and visual information to interact with the optokinetic (reviewed in Dieterich and Brandt, 2021). In summary, consistent with the interactions from the brainstem, there is a bilateral projection from the vestibular nuclei to the cortex, except for the lack of crossing fibers in the thalamus. The cortical areas innervated are the parietal insula, limbic and entorhinal cortex, and contacts the premotor cortical areas.

## Auditory nuclei depend on *Atoh1*

The expression of *Atoh1* in r2-5 (Wang et al., 2005) is needed to develop the anterior-ventral, posterior-ventral, and dorsal cochlear nuclei (AVCN, PVCN, and DCN; Figures 4A–C). Within dA1 is the expression of the bHLH gene *Olig3* and *Lhx2/9, Barhl1/2*, and *Pou4f1* to differentiate cells into neurons (Hernandez-Miranda et al., 2017). *Atoh1* expression depends on the choroid plexus formation to initiate *Lmx1a/b* to distinguish cochlear nuclei (Chizhikov et al., 2021). In addition, there is a loss of *Wnt3a* in *Lmx1a/b* double null mice (Elliott et al., 2021b). Furthermore, specific migration reaches out to add to dA1 and at least dA4 that depends on *Ptf1a* (Iskusnykh et al., 2016; Lipovsek et al., 2017; Hirsch et al., 2021). Cochlear nuclei are active before hair cells (Marrs and Spirou, 2012). Previous work showed that the SGN central projection typically develops in the absence of *Atoh1* and, thus, lacks hair cells and cochlear nuclei (Elliott et al., 2017). Future differentiation will generate a large set of cochlear nuclei that must be traced from the earliest gene expression to establish into bushy, globular, spherical, stellate, and pyramidal neurons (Malmierca, 2015; Oertel and Cao, 2020). In addition, unipolar brush cells, granule cells, and fusiform cells develop in the DCN; each is uniquely positive for calbindin, calretinin, and parvalbumin (reviewed in Caspary and Llano, 2018; Elliott et al., 2022b).

In addition to the cochlear nuclei (Figures 5A–C), several neurons migrate more ventrally to develop mostly from r4 to generate the medial olivocochlear nuclei (MOCs; Farago et al., 2006; Di Bonito et al., 2017). Different projections innervate ipsi- and contralateral MOCs (Marrs et al., 2013; Lipovsek and Wingate, 2018; Kandler et al., 2020; Grothe, 2021). For example, the development of detailed innervation shows an exuberant innervation of the MNTB followed by the development of the calyx of Held with time (Holcomb et al., 2013) that may decline in density formation with age (Elliott et al., 2022b). SGN fibers end up in a tonotopic organization that follows a base to apex progression to the MOCs. Both time and intensity are used to identify the unique frequency for future interactions (Grothe, 2021; Kandler et al., 2021). Innervation shows a bilateral projection from each cochlear nuclei to reach the lateral lemniscus to extend to the inferior colliculi (Macova et al., 2019; Syka, 2020). In summary (Figure 5), *Atoh1* is developing the three cochlear nuclei and designing the MOCs into distinct innervation to create massive calyx formations.

### Inferior colliculi and medial geniculate bodies project to the auditory cortex

Ascending projections to the inferior colliculi (IC) are bilateral projections either directly from the cochlear nucleus or indirectly from the cochlear nuclei (AVCN, PVCN, and DCN) through the MOCs and the lateral lemniscus, all while maintaining the tonotopic organization (Malmierca, 2015; Macova et al., 2019; Syka, 2020). The IC projects from the unilateral medial geniculate ganglion (MGB) that have limited crossing fibers in the IC (Grothe, 2021). Limited data (Gurung and Fritzsch, 2004; Razak et al., 2009) shows the earliest projection that has a prominent expression of *Neurod1* to the IC to project to the auditory nucleus (Malone et al., 2021). The central part of the IC is organized in a tonotopic organization that responds to both direct and indirect fibers (Malmierca, 2015; Budinger, 2020).

The MGB consists of several subareas, the major parts being the ventral (MGV), medial (MGM), and dorsal (MGD) divisions (Budinger, 2020). Its main ascending input, from the ventral nucleus of the medial geniculate body, provides connections that comprise only a fraction of the inputs to the auditory cortex (A1; ∼18%), where these ascending signals are integrated with feedback connections from ipsilateral (∼70%) and contralateral (∼10%) cortex (Malone et al., 2021). The MGV constitutes the tonotopic lemniscal part of the MGB (Figure 5A). The MGD and MGM are usually assigned to the largely non-tonotopically organized, non-lemniscal position (Malmierca, 2015; Budinger, 2020). MGB and IC receive direct input from the auditory cortex to influence information processing (Budinger, 2020; King, 2020). In summary, a bilateral relay from the IC is mostly unilateral to the MGB, which expands bilaterally in the auditory cortex.

## Integration of sensory inputs

Within the cortex, inputs from the various sensory systems interact at a tertiary level with inputs from other systems to provide an integrated sensory experience. Cortical integration of somatosensory, taste, vestibular, and auditory systems with other sensory and motor systems will be discussed below. Unfortunately, much of the detailed description is presented in humans, with a few exceptions from other mammals. Moreover, the detailed description will require additional analysis to evaluate the distinct genes needed for the integrated perspective.

### Somatosensory integration

**FIGURE 6:**
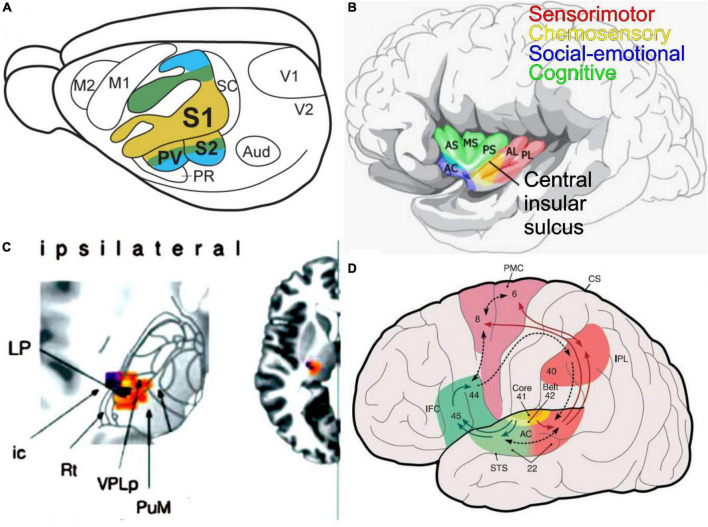
An integrated perspective of fur sensory inputs is presented. **(A)** Primary somatosensory (S1) has a topological projection that duplicates the secondary fibers (S2, PV)—taken from (Liao et al., 2020). **(B)** Within the insula are the sensorimotor, chemosensory, social-emotional, and cognitive interactions. AC, accessory gyrus; AL, anterior long insular gyrus; AS, anterior short insular gyrus; MS, middle short insular gyrus; PL, posterior long insular gyrus; PS, posterior short insular gyrus; VPPC, ventral posterior thalamic nucleus parvocellular. It was modified after (Zhang M. et al., 2020). **(C)** The ipsilateral inputs are shown to reach out *via* VPL to the cortex (insertion right). Modified after (Dieterich and Brandt, 2021). **(D)** The auditory cortex (AC, area 41, 42, 22) provides output to Brocca’s area (inferior frontal cortex, IFC; area 45, 46) for speech production, where it interacts with the premotor cortex (PMC, are 6, 8). Interaction is provided from the inferior parietal cortex (IPL, area 40) to interact with the superior temporal lobe (STS, area 22). Numbers provided by Brodmann. Taken from Rauschecker and Scott (2009).

Cortical *somatosensory* projections interact tertiarily with those from other sensory systems to form a cohesive perception (Hans et al., 2020). For example, multisensory integration depends on auditory, visual, olfactory, and somatosensory interaction (Meehan et al., 2017). Somatosensory neurons show integration properties like those of visual stimuli. Neural responses account for the perceived direction of the stimuli across all stimulus conditions tested, highlighting the somatosensory system, a vector average mechanism to compute tactile motion direction that bears striking similarities to its visual counterpart (Pei et al., 2011; Goodman and Bensmaia, 2021). In addition, the vibrissa information from the primary motor cortex (M1) modulates sensory processing in the primary somatosensory barrel cortex to provide interneurons *in vivo* that of somatostatin interneurons decreased during whisking, suggesting the firing rates during whisking depended on M1 activity (Figure 6A). A circuitry exists by which inputs from the motor cortex influence activity in the somatosensory cortex (Lee et al., 2013).

A secondary somatosensory area (S2) and the ventral parietal area (PV) are organized mirror-symmetrically relative to the primary somatosensory area in humans. Bilateral spatiotemporal integration in S2/PV takes place over a sizeable cortical area and presents the properties of spatiotemporal integration in anterior parietal regions. Importantly, S2/PV showed significant temporal integration while spatial integration was reduced (Zhu et al., 2007; Caldwell et al., 2019).

A tactile and visual interactive response to activation of various brain regions shows the effects of visuotactile multisensory integration on the amount of brain activation in the somatosensory cortical regions. Tactile stimulation-induced cortical activations occur in the left primary sensory-motor cortex (SM1) and secondary somatosensory cortex (S2). In the visuotactile stimulation task, cortical activations can happen in both primary S1 and secondary S2 and can reach the posterior parietal cortex by activation (Kwon et al., 2017). A visual and somatosensory input is reliable, reducing reliance on the vestibular system. Postural training can alter sensory organization after a visual feedback-vestibular activation training protocol, suggesting a possible sensory reweighting through vestibular adaptation and/or habituation occurs (Appiah-Kubi and Wright, 2019) that helps indicate with the somatosensory input, in particular the tendon of the limbs in humans (Peterka and Loughlin, 2004). Multisensory work in rodents suggested a segregation of mechanically driven, temperature-selective and gustatory responses that provide stimuli in the oral somatosensory cortex (Clemens et al., 2018). Furthermore, the mid-dorsal insula and the primary and secondary somatosensory cortex suggest that it involves a specific set of brain regions of the oral-sensory integration processes (Wistehube et al., 2018).

Beyond a hierarchical model of multisensory integration, a unimodal input converges onto higher multisensory areas that integrate multimodal information and guide decision-making and behavior. The superior temporal sulcus, lateral occipital–temporal cortex, posterior parietal cortex, and ventrolateral frontal cortex have been implicated in higher cortical multisensory processing (Meehan et al., 2017; Zhou et al., 2019). The involvement of primary sensory areas in multisensory processing indicates a multisensory integration that may involve more distributed neural networks beyond classic hierarchical multisensory-specific areas, including primary sensory cortices of the somatosensory input, including the associated somatosensory information (Brunert and Rothermel, 2020).

#### Somatosensory-auditory integrations

In the mouse, primary (S1) and secondary (S2) somatosensory cortices pair sound with whisker stimulation, modulating tactile responses in both S1 and S2. After local mutually inhibitory S2 circuit can spectrally select tactile versus auditory inputs suggesting a multisensory integration that provides a key role for S2 in combining auditory and tactile information (Zhang M. et al., 2020), Magnetoencephalography can be used to study auditory stimulation by cortical activity in the primary somatosensory cortex. Somatosensory–acoustic interaction reaches area 3b from the thalamus during the early stages of synaptic transmission (Sugiyama et al., 2018).

#### Somatosensory-vestibular interactions

Vestibular stimulation can speed tactile detection and indicates vestibular facilitation of somatosensory processing (Pfeiffer et al., 2018). Specifically, somatosensory- vestibular interactions affect the perceived timing of tactile stimuli (Moro and Harris, 2018). Caloric vestibular stimulation can result in somatosensory stimulation (Bretas et al., 2020) that can be combined with vestibular and postural training to improve balance in healthy young adults (Appiah-Kubi and Wright, 2019; Ferrè and Haggard, 2020). Area 3a extends from the somatosensory cortex and reaches the superior parietal lobule of the parietal-insula vestibular area (PIVC), and the extrastriate regions (Shaikh et al., 2020).

#### Somatosensory-taste interactions

Cross-modal correspondences occur between tastes and the somatosensory attributions of food and beverage products (Spence and Ngo, 2012). Beyond cross-modal integration occurs with somatosensory with sweet tastes (Spence, 2022). Results show that flavor perception must affect somatosensory stimuli as components of flavor perception and taste modulators (Green and Nachtigal, 2012). In mice, ascending projections from trigeminal neurons reach the parabrachial nucleus (PB). Trigeminal fibers use TRPV1 and TRPA1 to play a role in thermosensation and pain communication with parabrachial taste neurons (Li et al., 2021; Rhyu et al., 2021). In summary, mammals’ somatosensory cortical regions (S1, S2, and PV) have integrated with the motor cortex and visual, auditory, vestibular, taste, and olfactory cortical areas.

##### Conclusion of somatosensory inputs

Trigeminal input (Figures 2A, 6A) spans the spinal cord through the hindbrain to organize a topological input (leg > arm > face). Fibers reach the medial lemniscus, which projects to the VPL/VPM to innervate the principal (S1) and secondary somatosensory fibers (S2, PV). A tertiary interaction is evident in the motor cortex and has visual, auditory, vestibular, olfactory, and taste interactions to generate a cohesive perception. Further work is needed to detail the various sensory inputs to coordinate with the trigeminal primary (S1) and secondary (S2, PV) into a cohesive interaction.

### Taste integration

The solitary tract (ST) innervates, directly and indirectly, the parabrachial nuclei (PB, Figure 3A), from which primarily ipsilateral fibers extend the thalamus in mice (Lundy and Norgren, 2015; Gasparini et al., 2021). Neurons also directly reach the thalamus from ST to reach the gustatory and taste relay, referred to as ventral posterior thalamic nucleus parvocellular (VPPC). The unique ipsilateral connection and, to a lesser extent, the contralateral PB reach the insular cortex, which develops a reciprocal connection with gustatory and visceral afferents. The insula functions from sensory processing to representing feelings and emotions, autonomical and motor control, risk prediction and decision-making, bodily- and self-awareness, and complex social functions like empathy (Gogolla, 2017). Moreover, the insula subserves various human functions ranging from sensory and affective processing to high-level cognition (Uddin et al., 2017).

Beyond function, the loss of the right insula affects ipsilateral taste recognition, whereas the left-hemispheric stroke patients’ taste recognition on the right side of the tongue suggests that taste information from both sides of the tongue passes through the left insula (Pritchard et al., 1999). The insular cortex may be organized into a hedonic or “viscerotropic” map rather than one ordered according to taste quality. In contrast to a spatial map ordered by taste quality, the insular cortex interacts with other sensory inputs, integrating taste, somatosensation, and olfaction into a cohesive experience of food intake (Shepherd, 2006; Schier and Spector, 2019). Once it reaches the mouth, the taste, texture, and retronasal smell of the food deliver sensations of olfaction and taste (Figure 5B). The cortical presentation of both spatial and temporal divisions of taste coding changes with experience. Cortical taste representation is not organized in a spatially discrete map but instead is plastic and spatially dispersed that uses temporal information to encode many types of ingested stimuli (Staszko et al., 2020).

In contrast to visual, auditory, and somatosensory cortices that are topographically organized, the spatial organization of taste responses in the gustatory insular cortex responses shows no sign of spatial clustering or topography. Thus, the idea that taste qualities in the insula are sparse and distributed is analogous to the representation of odorants in the piriform cortex (Chen et al., 2021). In humans, recent studies do not support the topographic model of taste quality representation, but rather taste quality is a distributed pattern within gustatory regions of the insula (Avery, 2021). Several indications suggest that information about taste stimuli is more profound when paired with odors than when presented alone (Lorenzo, 2021). These data show that a lack of direct projections from the olfactory bulb or piriform cortex may be conveyed *via* the gustatory cortex, amygdala, or lateral hypothalamus, suggesting a convergence of information of olfaction and taste (Lorenzo, 2021).

Four functionally distinct regions have been identified in the human insula (Figure 5B): (1) a sensorimotor region located in the mid-posterior insula; (2) a central olfactory-gustatory region; (3) a socio-emotional region in the anterior-ventral insula; and (4) a cognitive anterior-dorsal region (Uddin et al., 2017). Trigeminal and olfactory input converges and integrates within the insular region (Hummel et al., 2009) which is expanded for spicy stimulations in humans (Han et al., 2021; Spence et al., 2021). Somatosensory manifestations represent a large proportion of responses elicited by electrical stimulation of the insular cortex in humans. Likewise, input from the auditory system and the parieto-insular vestibular cortex reach the insular cortex. The posterior granular insula in monkeys integrates vestibular inputs and proprioceptive, visual motion, and auditory activities comparable to humans (Evrard, 2019). However, an interaction between auditory, speech influence, and vestibular integration with taste input is unclear (Uddin et al., 2017). A poly-modal integration likely contributes to “higher” vestibular functions, including self-motion perception in a body- (proprioceptive) and the world- (audio-visual) centered referential system that could be organized into a dorsoventral gradient (Evrard, 2019).

#### Taste-auditory integrations

Tasting in the presence of auditory stimuli of various frequencies or silence suggests that low frequencies increase red wine’s perception and aromatic intensity (Crisinel and Spence, 2009; Burzynska et al., 2019). Moreover, cross-modal interactions can affect the background music on the drink’s taste (Hauck and Hecht, 2019).

#### Taste-vestibular interactions

Vestibular stimulation can speed tactile detection and indicates vestibular facilitation of somatosensory processing (Pfeiffer et al., 2018). Caloric vestibular stimulation can result in somatosensory stimulation (Bretas et al., 2020) combined with vestibular and postural training (Appiah-Kubi and Wright, 2019; Ferrè and Haggard, 2020).

#### Taste-somatosensory interactions

Cross-modal correspondences between taste and the somatosensory attributions of food and beverage products (Spence and Ngo, 2012). Beyond cross-modal integration with somatosensory in particular, sweet flavors (Spence, 2022) that connect to drinking have unclear interactions (Green and Nachtigal, 2012).

##### Conclusion of taste inputs

The taste bud projection (Figures 3A, 6B) from the three visceral ganglia reaches r4-11 to form the solitary tract. Fibers reach from the ST to the PB and go bilaterally to the VPPC to innervate the insula. To generate a cohesive perception, a tertiary interaction is limited to somatosensory, olfactory, auditory, and vestibular interactions.

### Vestibular integration

In monkeys, the PIVC seems to be the dominant multi-modal vestibular cortical area, which is closely connected to the other regions, the vestibular nucleus, and to the opposite hemisphere (Figure 4A). Neurons are focused on VPL and ADN but have additional projections from MVN and SVN (Dieterich and Brandt, 2018). The thalamus is multimodal and encodes vestibular signals to project somatosensory, proprioceptive, visual, and auditory sensory information (Figure 4A). From here, the VPL is important as multisensory inputs to have a perceptual perception of head and body motion that are directly innervating the parietal-insula of the vestibular cortical area (PIVC) and the ventral intraparietal areas (VIP; Cullen, 2019). A strong connection exists with 3a and 2v that interacts with multiple connections to play a role in the frontal eye field. Notable inputs are the medial superior temporal area that plays a role in visual motion that interacts with vestibular and visual stimulation (Cullen, 2019). Without any doubt, the visual-vestibular interactions are directly and indirectly involved in optokinetic stimulation and caloric irrigation in normal and partial deletion of various regions (Dieterich and Brandt, 2019; Cullen et al., 2021). Two areas, the lateral entorhinal and postrhinal cortex contain center-bearing and center-distance cells (Figure 4C), which discharge based on the animal’s orientation and distance to the center of its environment (Taube and Yoder, 2021). Going beyond the visual/vestibular interactions, we provide an overview of somatosensory, taste, and auditory integration. Because this vestibular cortical network is so widely distributed, it could impact multiple neurocognitive functions in health and disease. The most impact is dependent on Caloric Vestibular Stimulation (CVS) or Galvanic Vestibular Stimulation (GVS) to cause an integrated perspective (Ferrè and Haggard, 2020).

#### Vestibular-somatosensory interactions

Following the peripersonal space can be used to define upper limbs for vestibular signals (Pfeiffer et al., 2018). For example, vestibular stimulation can speed tactile detection and indicates vestibular facilitation of somatosensory processing, suggesting that vestibular inputs dynamically interact with multisensory input (Pfeiffer et al., 2018). Caloric vestibular stimulation can result in somatosensory activity (Bretas et al., 2020) that can be activated after somatosensory reduction and can combine vestibular and postural training to improve balance in healthy young adults and likely in those with deficits (Appiah-Kubi and Wright, 2019; Ferrè and Haggard, 2020).

#### Vestibular-taste interactions

Pairing a novel taste with an offensive vestibular stimulation result in conditioned taste aversions in rats and humans. Vestibular system involvement in gustatory conditioning was compared in sham-lesioned or labyrinth ectomized rats with control rats for oral rejection reactions (Ossenkopp et al., 2003). Previous work showed that vestibular input could influence several aspects of consumer behavior to affect food taste (Biswas et al., 2019). Robust taste aversions can be solicited in rats and mice affected after the bilateral loss of labyrinth function, suggesting a direct interaction of the magnet on the vestibular system in *Het* mutant mice (Cote, 2020).

#### Vestibular-auditory interactions

Rhythm can be biased by passive motion, which suggests that vestibular input may play a vital role in the effect of movement on auditory rhythm processing (Phillips-Silver and Trainor, 2008; Phillips-Silver et al., 2020). Recent evidence suggests that simultaneous auditory–vestibular training facilitates short-term auditory plasticity, producing stronger oscillator connections in the acoustic network (Tichko et al., 2021). Most importantly, a combined vestibular and cochlear prosthesis may restore hearing and balance in patients who have lost both (Phillips et al., 2020). A significant interaction between different sensory modalities during stimulation with a combined vestibular and cochlear prosthesis can help challenge stimulation strategies to simultaneously restore auditory and vestibular function after such an implant (Phillips et al., 2020).

##### Conclusion of vestibular inputs

Vestibular information (Figures 4A, 6C) is sent from the periphery to four vestibular nuclei (spanning r1-r8) along vestibular sensory neurons. Ascending projections are bilateral to innervate the ventral posterolateral nucleus (VPL) and anterior vestibular-thalamic pathway (ADN) to the parietal insula of the vestibular cortical area (PIVC) and the ventral intraparietal regions (VIP). Higher-order integration beyond the demonstrated visual-vestibular interaction is limited to somatosensory, taste, and, importantly, auditory-vestibular interactions.

### Auditory integration

The auditory cortex (A1) is a central hub located at a pivotal position within the auditory system, receiving a tonotopic input from the medial geniculate body (MGB) and playing a role in the sensation, perception (Figure 5A), and interpretation of the acoustic environment (Malone et al., 2021). ‘Receptive fields’ (RF) end up a particular stimulus dimension: the choice of acoustic stimulus dimensions to explore are related to the basic perceptual attributes such as spectral pitch (frequency), loudness (intensity), periodicity, or virtual pitch (amplitude modulations, harmonic series), timbre (spectral envelope), and sound location (interaural time, level differences, and spectral shape). In humans, hemispheric asymmetries between the left and suitable auditory cortex form in gross anatomical features and cortical microcircuitry (Budinger, 2020; Rauschecker, 2021).

There are two major classes of neurons in the neocortex: principal cells and interneurons. The auditory cortex is organized into six horizontal layers, another defining feature of the neocortex. It has anatomical and functional vertical columns and has intense interhemispheric connections between the auditory cortices of both hemispheres, which cross the midline *via* the (caudal body of the) corpus callosum (Budinger, 2020; Malone et al., 2021). Primary sensory cortices like A1, S1 (somatosensory), and V1(visual) are not purely unimodal but also process other non-matched sensory and non-sensory information. Projections from V1 and, to a lesser degree, from S1 toward A1 mainly arise from subgranular layers and provide feedforward organizations (Budinger, 2020).

The auditory corticobulbar system includes projections from the nuclei of the medial geniculate body (MGB), the inferior colliculi (IC), the lateral lemniscus (NLL), superior olivary complex (SOC), and the cochlear nuclear complex (CN) including the medial olivary efferents that project out to the outer hair cells (OHCs; Simmons et al., 2011; King, 2020). The prominent corticothalamic, corticocollicular, colliculofugal, and olivocochlear connections descend from auditory pathways that include long-range connections (Malmierca, 2015; Budinger, 2020; King, 2020). Overall, the feedback loop might modulate auditory response properties of neurons in the midbrain and hindbrain, adapting their sensitivity to sound frequency, intensity, and location (Budinger, 2020; King, 2020).

As previously mentioned, the auditory cortex interacts with multiple other sensory projections as tertiary fibers (Figure 5D). For example, auditory soundscape or visual landscape can influence the perception in a real, multisensory environment (Pheasant et al., 2010; Zhao et al., 2018). An interesting McGurke effect showed an interaction between visual and auditory stimuli, highlighting a higher interaction of otherwise discrete stimulations (Spence and Soto-Faraco, 2010). Most interestingly, multisensory interactions are audiovisual speech perception, in which visual speech substantially enhances auditory speech processes (Plass et al., 2020) that be more robust in audio-visual speech responses than what would have been expected from the summation of the audio and visual speech responses, suggesting that multisensory integration occurs (O’Sullivan et al., 2021).

#### Auditory-somatosensory interactions

Typical, auditory-somatosensory (AS) humans can multisensory stimulus pairs yield significant reaction time facilitation relative to their unisensory counterparts that exceeded probability (Murray et al., 2005). Multisensory interactions across space suggest perceptual-cognitive phenomena of sensory-cognitive processing (Foxe et al., 2002). Further, integration of auditory and somatosensory information in speech processing can be used in a bimodal perceptual task, suggesting somatosensory information on sound categorization can affect adults more than children (Trudeau-Fisette et al., 2019). This results in somatosensory cross-modal reorganization of the auditory cortex in adults with early stage, mild-moderate age-related hearing loss [ARHL (Cardon and Sharma, 2018)]. Pairing sound with whisker stimulation modulates tactile responses in both S1 and S2, with the most prominent modulation being robust inhibition in S2 (Zhang M. et al., 2020). Mutually inhibitory activation from the S2 circuit can spectrally select tactile versus auditory inputs in mice (Zhang M. et al., 2020).

#### Auditory-taste interactions

In contrast to vision and somatosensory interaction, very few examples exist supporting some level of interaction between the auditory and taste systems (Keast and Breslin, 2003; Zampini and Spence, 2012). The strongest is provided through a cross interaction between taste and auditory pitch (Holt-Hansen, 1968; Spence, 2011). Most recent evidence suggests an attenuation of taste neophobia induced by taste familiarity and is auditory context-dependent in mice (Grau-Perales and Gallo, 2020). Context dependency involved dopaminergic activity mediated by D1 receptors, which might be responsible for adequately acquiring safe taste recognition memory (Grau-Perales et al., 2021). In humans, associations between auditory attributes and a number of the commonly agreed basic tastes are now recently added as ‘saltiness’ (Wang et al., 2021), among other interactions of taste with auditory interactions (Spence et al., 2021).

#### Auditory-vestibular interactions

Since rhythm can be biased by passive motion, it suggests that vestibular input may play a vital role in the effect of movement on auditory rhythm processing (Phillips-Silver and Trainor, 2008; Phillips-Silver et al., 2020). Recent evidence suggests that simultaneous auditory-vestibular training facilitates short-term auditory plasticity, producing stronger oscillator connections in the auditory network (Tichko et al., 2021). Most importantly, it can be combined in a vestibular and cochlear prosthesis to restore hearing and balance in patients who have lost both (Phillips et al., 2020). A significant interaction between different sensory modalities during stimulation with a combined vestibular and cochlear prosthesis can help challenge stimulation strategies to simultaneously restore auditory and vestibular function after such an implant (Phillips et al., 2020).

##### Conclusion on auditory integration

Cochlear spiral ganglia (Figures 5A, 6D) are unique compared to the nearby vestibular ganglia and project tonotopically to innervate the cochlear nuclei that form from r2-5. A notable development migrates from the cochlear nuclei (r4) to receive order projections. Projections are bilateral to innervate *via* IC and MGB to innervate the A1. Higher-order auditory interactions are limited to somatosensory, taste, and, importantly, the auditory-vestibular interactions.

## A combined perspective of the four brainstem sensory systems

The peripheral sensory innervation depends on unique regional cell types (Merkel cells, free endings, taste buds, and mechanosensory hair cells). Trigeminal, vestibular, auditory and taste neurons depend on the bHLH genes, *Neurog1/2*. Central organization depends on *Atoh1* (dA1) for auditory, *Phox2b/Tbx3* (dA3) for taste, *Phox2b/Ptf1a* (dB1/2) for vestibular and *Ascl1/Neurog1* (dB3/4) for trigeminal to integrate through migration into the distinct brainstem organization. Output is prominent mainly to the contralateral thalamus (trigeminal), ipsilateral *via* an intermediate (PB, taste) to the thalamus, bilaterally through the midbrain intermediate to the thalamus in the vestibular, and bilaterally from the auditory a secondary (SOC, LL) to the tertiary (IC) and the thalamus (MGB). From here, a topological organization projects to the somatosensory (S1) and the auditory telencephalon (A1), whereas the taste (insula) and vestibular (VPL, PIVC) are not organized topologically. Higher-order interactions are demonstrated between trigeminal and auditory, between taste and vestibular, between the auditory and the vestibular, and between taste and trigeminal to integrate the cohesive perception of the telencephalon mostly documented in human.

## Data availability statement

The original contributions presented in this study are included in the article/supplementary material, further inquiries can be directed to the corresponding author.

## Author contributions

All authors listed have made a substantial, direct, and intellectual contribution to the work, and approved it for publication.

## References

[B1] Abraira V. E. Ginty D. D. (2013). The sensory neurons of touch. *Neuron* 79 618–639. 10.1016/j.neuron.2013.07.051 23972592 PMC3811145

[B2] Alsina B. (2020). Mechanisms of cell specification and differentiation in vertebrate cranial sensory systems. *Curr. Opin. Cell Biol.* 67 79–85. 10.1016/j.ceb.2020.08.006 32950922

[B3] Andrew D. L. E. May P. J. Warren S. (2020). Morphologic characterization of trigeminothalamic terminal arbors arising from the principal nucleus in the macaque. *Front. Neuroanat.* 14:562673. 10.3389/fnana.2020.562673 33041774 PMC7525072

[B4] Antón-Bolaños N. Sempere-Ferràndez A. Guillamón-Vivancos T. Martini F. J. Pérez-Saiz L. Gezelius H. (2019). Prenatal activity from thalamic neurons governs the emergence of functional cortical maps in mice. *Science* 364 987–990. 10.1126/science.aav7617 31048552 PMC7611000

[B5] Appiah-Kubi K. O. Wright W. (2019). Vestibular training promotes adaptation of multisensory integration in postural control. *Gait Posture* 73 215–220. 10.1016/j.gaitpost.2019.07.197 31376748

[B6] Arimura N. Dewa K. I. Okada M. Yanagawa Y. Taya S. I. Hoshino M. (2019). Comprehensive and cell-type-based characterization of the dorsal midbrain during development. *Genes Cells* 24 41–59. 10.1111/gtc.12656 30422377

[B7] Avery J. A. (2021). Against gustotopic representation in the human brain: There is no Cartesian restaurant. *Curr. Opin. Physiol.* 20 23–28. 10.1016/j.cophys.2021.01.005 33521413 PMC7839947

[B8] Avery J. A. Liu A. G. Ingeholm J. E. Riddell C. D. Gotts S. J. Martin A. (2020). Taste quality representation in the human brain. *J. Neurosci.* 40 1042–1052. 10.1523/JNEUROSCI.1751-19.2019 31836661 PMC6989007

[B9] Balmer T. S. Trussell L. O. (2019). Selective targeting of unipolar brush cell subtypes by cerebellar mossy fibers. *eLife* 8:e44964. 10.7554/eLife.44964 30994458 PMC6469928

[B10] Bermingham N. A. Hassan B. A. Price S. D. Vollrath M. A. Ben-Arie N. Eatock R. A. (1999). Math1: An essential gene for the generation of inner ear hair cells. *Science* 284 1837–1841. 10.1126/science.284.5421.1837 10364557

[B11] Bermingham N. A. Hassan B. A. Wang V. Y. Fernandez M. Banfi S. Bellen H. J. (2001). Proprioceptor pathway development is dependent on Math1. *Neuron* 30 411–422. 10.1016/s0896-6273(01)00305-1 11395003

[B12] Bigiani M. (2021). “Salt taste,” in *The senses*, ed. Fritzsch B. (Amsterdam: Elsevier), 247–263. 10.1016/B978-0-12-809324-5.23910-2

[B13] Birol O. Ohyama T. Edlund R. K. Drakou K. Georgiades P. Groves A. K. (2016). The mouse Foxi3 transcription factor is necessary for the development of posterior placodes. *Dev. Biol.* 409 139–151. 10.1016/j.ydbio.2015.09.022 26550799 PMC4688051

[B14] Biswas D. Szocs C. Abell A. (2019). Extending the boundaries of sensory marketing and examining the sixth sensory system: Effects of vestibular sensations for sitting versus standing postures on food taste perception. *J. Consum. Res.* 46 708–724. 10.1093/jcr/ucz018

[B15] Bouchard M. de Caprona D. Busslinger M. Xu P. Fritzsch B. (2010). Pax2 and Pax8 cooperate in mouse inner ear morphogenesis and innervation. *BMC Dev. Biol.* 10:89. 10.1186/1471-213X-10-89 20727173 PMC2939565

[B16] Bretas R. V. Taoka M. Suzuki H. Iriki A. (2020). Secondary somatosensory cortex of primates: Beyond body maps, toward conscious self-in-the-world maps. *Exp. Brain Res.* 238 259–272. 10.1007/s00221-020-05727-9 31960104 PMC7007896

[B17] Briscoe S. D. Ragsdale C. W. (2019). Evolution of the chordate telencephalon. *Curr. Biol.* 29 R647–R662. 10.1016/j.cub.2019.05.026 31287987 PMC11073819

[B18] Brunert D. Rothermel M. (2020). Cortical multisensory integration—a special role of the agranular insular cortex? *Pflügers Arch.* 472 671–672. 10.1007/s00424-020-02400-6 32458084 PMC7293668

[B19] Budinger E. (2020). “Primary auditory cortex and the thalamo-cortico-thalamic circuitry,” in *The senses*, ed. Fritzsch B. 10.1016/B978-0-12-805408-6.00022-1 (Amsterdam: Elsevier), 623–656.

[B20] Burzynska J. Wang Q. J. Spence C. Bastian S. E. P. (2019). Taste the bass: Low frequencies increase the perception of body and aromatic intensity in red wine. *Multisens. Res.* 32 429–454. 10.1163/22134808-20191406 31117049

[B21] Caldwell D. J. Cronin J. A. Wu J. Weaver K. E. Ko A. L. Rao R. P. (2019). Direct stimulation of somatosensory cortex results in slower reaction times compared to peripheral touch in humans. *Sci. Rep.* 9:3292. 10.1038/s41598-019-38619-2 30824821 PMC6397274

[B22] Canna A. Prinster A. Cantone E. Ponticorvo S. Russo A. G. Di Salle F. (2019). Intensity-related distribution of sweet and bitter taste fMRI responses in the insular cortex. *Hum. Brain Mapp.* 40 3631–3646. 10.1002/hbm.24621 31066980 PMC6865662

[B23] Cardon G. Sharma A. (2018). Somatosensory cross-modal reorganization in adults with age-related, early-stage hearing loss. *Front. Hum. Neurosci.* 12:172. 10.3389/fnhum.2018.00172 29773983 PMC5943502

[B24] Caspary D. M. Llano D. A. (2018). “Aging processes in the subcortical auditory system,” in *The oxford handbook of the auditory brainstem*, ed. Kandler K. (Oxford: Oxford University Press), 639–679.

[B25] Chagnaud B. P. Engelmann J. Fritzsch B. Glover J. C. Straka H. (2017). Sensing external and self-motion with hair cells: A comparison of the lateral line and vestibular systems from a developmental and evolutionary perspective. *Brain Behav. Evol.* 90 98–116. 10.1159/000456646 28988233 PMC5653922

[B26] Chen K. Kogan J. F. Fontanini A. (2021). Spatially distributed representation of taste quality in the gustatory insular cortex of behaving mice. *Curr. Biol.* 31 247–256.e4. 10.1016/j.cub.2020.10.014 33186554 PMC7855361

[B27] Chen X. Gabitto M. Peng Y. Ryba N. J. Zuker C. S. (2011). A gustotopic map of taste qualities in the mammalian brain. *Science* 333 1262–1266. 10.1126/science.1204076 21885776 PMC3523322

[B28] Chen Y. Takano-Maruyama M. Fritzsch B. Gaufo G. O. (2012). Hoxb1 controls anteroposterior identity of vestibular projection neurons. *PLoS One* 7:e34762. 10.1371/journal.pone.0034762 22485187 PMC3317634

[B29] Chessum L. Matern M. S. Kelly M. C. Johnson S. L. Ogawa Y. Milon B. (2018). Helios is a key transcriptional regulator of outer hair cell maturation. *Nature* 563 696–700. 10.1038/s41586-018-0728-4 30464345 PMC6542691

[B30] Chizhikov V. V. Iskusnykh I. Y. Fattakhov N. Fritzsch B. (2021). Lmx1a and Lmx1b are redundantly required for the development of multiple components of the mammalian auditory system. *Neuroscience* 452 247–264. 10.1016/j.neuroscience.2020.11.013 33246067 PMC7780644

[B31] Chou X. L. Fang Q. Yan L. Zhong W. Peng B. Li H. (2020). Contextual and cross-modality modulation of auditory cortical processing through pulvinar mediated suppression. *eLife* 9:e54157. 10.7554/eLife.54157 32142411 PMC7080503

[B32] Clemens A. M. Delgado Y. F. Mehlman M. L. Mishra P. Brecht M. (2018). Multisensory and motor representations in rat oral somatosensory cortex. *Sci. Rep.* 8:13556. 10.1038/s41598-018-31710-0 30201995 PMC6131144

[B33] Cote J. (2020). Het mouse model suggests vestibular system mediates magnetic field effects. *Biophys. J.* 118:613a. 10.1016/j.bpj.2019.11.3313

[B34] Crisinel A.-S. Spence C. (2009). Implicit association between basic tastes and pitch. *Neurosci. Lett.* 464 39–42. 10.1016/j.neulet.2009.08.016 19679162

[B35] Cullen K. E. (2019). Vestibular processing during natural self-motion: Implications for perception and action. *Nat. Rev. Neurosci.* 20 346–363. 10.1038/s41583-019-0153-1 30914780 PMC6611162

[B36] Cullen K. E. Zobeini O. A. Woboonsaksakul K. P. Wang L. Stanley O. R. Leavitt O. M. E. (2021). “Self-motion,” in *The senses*, ed. Fritzsch B. 10.1016/B978-0-12-809324-5.24178-3 (Amsterdam: Elsevier), 483–495.

[B37] Daghfous G. Auclair F. Blumenthal F. Suntres T. Lamarre-Bourret J. Mansouri M. (2020). Sensory cutaneous papillae in the sea lamprey (*Petromyzon marinus* L.): I. Neuroanatomy and physiology. *J. Comp. Neurol.* 528 664–686. 10.1002/cne.24787 31605382

[B38] D’Autréaux F. Coppola E. Hirsch M. R. Birchmeier C. Brunet J. F. (2011). Homeoprotein Phox2b commands a somatic-to-visceral switch in cranial sensory pathways. *Proc. Natl. Acad. Sci. U.S.A.* 108 20018–20023. 10.1073/pnas.1110416108 22128334 PMC3250195

[B39] Dennis D. J. Han S. Schuurmans C. (2019). bHLH transcription factors in neural development, disease, and reprogramming. *Brain Res.* 1705 48–65. 10.1016/j.brainres.2018.03.013 29544733

[B40] Desai S. S. Zeh C. Lysakowski A. (2005). Comparative morphology of rodent vestibular periphery. I. Saccular and utricular maculae. *J. Neurophysiol.* 93, 251–266. 15240767 10.1152/jn.00746.2003 PMC12456082

[B41] Di Bonito M. Studer M. Puelles L. (2017). Nuclear derivatives and axonal projections originating from rhombomere 4 in the mouse hindbrain. *Brain Struct. Funct.* 222 3509–3542. 10.1007/s00429-017-1416-0 28470551 PMC5676809

[B42] Diaz C. Glover J. C. (2021). The vestibular column in the mouse: A rhombomeric perspective. *Front. Neuroanat.* 15:806815. 10.3389/fnana.2021.806815 35173589 PMC8842660

[B43] Dieterich M. Brandt T. (2018). Global orientation in space and the lateralization of brain functions. *Curr. Opin. Neurol.* 31 96–104. 10.1097/WCO.0000000000000516 29189299

[B44] Dieterich M. Brandt T. (2019). Perception of verticality and vestibular disorders of balance and falls. *Front. Neurol.* 10:172. 10.3389/fneur.2019.00172 31001184 PMC6457206

[B45] Dieterich M. Brandt T. (2021). “Structural and functional imaging of the human bilateral vestibular network from the brainstem to the cortical hemispheres,” in *The senses*, ed. Fritzsch B. (Amsterdam: Elsevier), 414–431. 10.1016/B978-0-12-809324-5.23886-8

[B46] Dieterich M. Kirsch V. Brandt T. (2017). Right-sided dominance of the bilateral vestibular system in the upper brainstem and thalamus. *J. Neurol.* 264 55–62. 10.1007/s00415-017-8453-8 28315957

[B47] Duncan J. S. Cox B. C. (2020). “Anatomy and development of the inner ear,” in *Reference module in neuroscience and biobehavioral psychology*, ed. Stein J. 10.1016/B978-0-12-809324-5.24161-8 (Amsterdam: Elsevier).

[B48] Duncan J. S. Fritzsch B. (2013). Continued expression of GATA3 is necessary for cochlear neurosensory development. *PLoS One* 8:e62046. 10.1371/journal.pone.0062046 23614009 PMC3628701

[B49] Duncan J. S. Fritzsch B. Houston D. W. Ketchum E. M. Kersigo J. Deans M. R. (2019). Topologically correct central projections of tetrapod inner ear afferents require Fzd3. *Sci. Rep.* 9:10298. 10.1038/s41598-019-46553-6 31311957 PMC6635624

[B50] Dvorakova M. Macova I. Bohuslavova R. Anderova M. Fritzsch B. Pavlinkova G. (2020). Early ear neuronal development, but not olfactory or lens development, can proceed without SOX2. *Dev. Biol.* 457 43–56. 10.1016/j.ydbio.2019.09.003 31526806 PMC6938654

[B51] Dvoryanchikov G. Hernandez D. Roebber J. K. Hill D. L. Roper S. D. Chaudhari N. (2017). Transcriptomes and neurotransmitter profiles of classes of gustatory and somatosensory neurons in the geniculate ganglion. *Nat. Commun.* 8:760. 10.1038/s41467-017-01095-1 28970527 PMC5624912

[B52] Elliott K. L. Sokolowski B. Yamoah E. N. Fritzsch B. (2022a). *An integrated perspective of commonalities and differences across sensory receptors and their distinct inputs.* 10.1201/9781003092810-10 Boca Raton, FL: Wiley.

[B53] Elliott K. L. Fritzsch B. Yamoah E. N. Zine A. (2022b). Age-related hearing loss: Sensory and neural etiology and their interdependence. *Front. Aging Neurosci.* 14:814528. 10.3389/fnagi.2022.814528 35250542 PMC8891613

[B54] Elliott K. L. Gordy C. (2020). “Ear vestibular neurosensory development,” in *The senses*, ed. Fritzsch B. 10.1016/B978-0-12-809324-5.24141-2 (Amsterdam: Elsevier), 145–161.

[B55] Elliott K. L. Kersigo J. Lee J. H. Jahan I. Pavlinkova G. Fritzsch B. (2021a). Developmental changes in peripherin-eGFP expression in spiral ganglion neurons. *Front. Cell. Neurosci.* 15:678113. 10.3389/fncel.2021.678113 34211371 PMC8239239

[B56] Elliott K. L. Pavlínková G. Chizhikov V. V. Yamoah E. N. Fritzsch B. (2021b). Development in the mammalian auditory system depends on transcription factors. *Int. J. Mol. Sci.* 22:4189. 33919542 10.3390/ijms22084189 PMC8074135

[B57] Elliott K. L. Kersigo J. Pan N. Jahan I. Fritzsch B. (2017). Spiral ganglion neuron projection development to the hindbrain in mice lacking peripheral and/or central target differentiation. *Front. Neural Circuits* 11:25. 10.3389/fncir.2017.00025 28450830 PMC5389974

[B58] Elliott K. L. Straka H. (2022). *Assembly and functional organization of the vestibular system*, eds Fritzsch B. Elliott K. L. 10.1201/9781003092810-6 (Boca Raton, FL: Wiley), 135–174.

[B59] Eng S. R. Dykes I. M. Lanier J. Fedtsova N. Turner E. E. (2007). POU-domain factor Brn3a regulates both distinct and common programs of gene expression in the spinal and trigeminal sensory ganglia. *Neural Develop.* 2:3. 10.1186/1749-8104-2-3 17239249 PMC1796875

[B60] Erives A. Fritzsch B. (2020). A screen for gene paralogies delineating evolutionary branching order of early metazoa. *G3* 10 811–826. 10.1534/g3.119.400951 31879283 PMC7003098

[B61] Erzurumlu R. S. Gaspar P. (2020). How the barrel cortex became a working model for developmental plasticity: A historical perspective. *J. Neurosci.* 40 6460–6473. 10.1523/JNEUROSCI.0582-20.2020 32817388 PMC7486654

[B62] Erzurumlu R. S. Murakami Y. Rijli F. M. (2010). Mapping the face in the somatosensory brainstem. *Nat. Rev. Neurosci.* 11 252–263. 10.1038/nrn2804 20179712 PMC3545448

[B63] Evrard H. C. (2019). The organization of the primate insular cortex. *Front. Neuroanat.* 13:43. 10.3389/fnana.2019.00043 31133822 PMC6517547

[B64] Fain G. L. (2019). *Sensory transduction.* 10.1093/oso/9780198835028.001.0001 Oxford: Oxford University Press.

[B65] Fan D. Chettouh Z. Consalez G. G. Brunet J.-F. (2019). Taste bud formation depends on taste nerves. *eLife* 8:e49226. 10.7554/eLife.49226.017 31570121 PMC6785267

[B66] Farago A. F. Awatramani R. B. Dymecki S. M. (2006). Assembly of the brainstem cochlear nuclear complex is revealed by intersectional and subtractive genetic fate maps. *Neuron* 50 205–218. 10.1016/j.neuron.2006.03.014 16630833

[B67] Ferrè E. R. Haggard P. (2020). Vestibular cognition: State-of-the-art and future directions. *Cogn. Neuropsychol.* 37 413–420. 10.1080/02643294.2020.1736018 32192411

[B68] Filova I. Dvorakova M. Bohuslavova R. Pavlinek A. Elliott K. L. Vochyanova S. (2020). Combined Atoh1 and Neurod1 deletion reveals autonomous growth of auditory nerve fibers. *Mol. Neurobiol.* 57 5307–5323. 10.1007/s12035-020-02092-0 32880858 PMC7547283

[B69] Finger T. E. (1978). Gustatory pathways in the bullhead catfish. II. Facial lobe connections. *J. Comp. Neurol.* 180 691–705. 10.1002/cne.901800404 79574

[B70] Finger T. E. (2008). Sorting food from stones: The vagal taste system in Goldfish, *Carassius auratus*. *J. Comp. Physiol. A* 194 135–143. 10.1007/s00359-007-0276-0 18228077 PMC2543128

[B71] Fode C. Gradwohl G. Morin X. Dierich A. LeMeur M. Goridis C. (1998). The bHLH protein NEUROGENIN 2 is a determination factor for epibranchial placode–derived sensory neurons. *Neuron* 20 483–494. 10.1016/s0896-6273(00)80989-7 9539123

[B72] Foxe J. J. Wylie G. R. Martinez A. Schroeder C. E. Javitt D. C. Guilfoyle D. (2002). Auditory-somatosensory multisensory processing in auditory association cortex: An fMRI study. *J. Neurophysiol.* 88 540–543. 10.1152/jn.2002.88.1.540 12091578

[B73] Freter S. Muta Y. O’Neill P. Vassilev V. S. Kuraku S. Ladher R. K. (2012). Pax2 modulates proliferation during specification of the otic and epibranchial placodes. *Dev. Dyn.* 241 1716–1728. 10.1002/dvdy.23856 22972769

[B74] Fritzsch B. (2021). An integrated perspective of evolution and development: From genes to function to ear, lateral line and electroreception. *Diversity* 13:364. 10.3390/d13080364 35505776 PMC9060560

[B75] Fritzsch B. Beisel K. Jones K. Farinas I. Maklad A. Lee J. (2002). Development and evolution of inner ear sensory epithelia and their innervation. *J. Neurobiol.* 53 143–156. 10.1002/neu.10098 12382272 PMC4943216

[B76] Fritzsch B. Dillard M. Lavado A. Harvey N. L. Jahan I. (2010). Canal cristae growth and fiber extension to the outer hair cells of the mouse ear require Prox1 activity. *PLoS One* 5:e9377. 10.1371/journal.pone.0009377 20186345 PMC2826422

[B77] Fritzsch B. Elliott K. L. (2017). Gene, cell, and organ multiplication drives inner ear evolution. *Dev. Biol.* 431 3–15. 10.1016/j.ydbio.2017.08.034 28866362 PMC5643246

[B78] Fritzsch B. Elliott K. L. Glover J. C. (2017). Gaskell revisited: New insights into spinal autonomics necessitate a revised motor neuron nomenclature. *Cell Tissue Res.* 370 195–209. 10.1007/s00441-017-2676-y 28856468 PMC5641259

[B79] Fritzsch B. Elliott K. L. Pavlinkova G. (2019). Primary sensory map formations reflect unique needs and molecular cues specific to each sensory system. *F1000Res.* 8:F1000FacultyRev-345. 10.12688/f1000research.17717.1 30984379 PMC6439788

[B80] Fritzsch B. Martin P. R. (2022). Vision and retina evolution: How to develop a retina. *IBRO Neurosci. Rep.* 12 240–248. 35449767 10.1016/j.ibneur.2022.03.008 PMC9018162

[B81] Fritzsch B. Matei V. Nichols D. Bermingham N. Jones K. Beisel K. (2005). Atoh1 null mice show directed afferent fiber growth to undifferentiated ear sensory epithelia followed by incomplete fiber retention. *Dev. Dyn.* 233 570–583. 10.1002/dvdy.20370 15844198 PMC1242170

[B82] Fritzsch B. Northcutt G. (1993). Cranial and spinal nerve organization in amphioxus and lampreys: Evidence for an ancestral craniate pattern. *Cells Tissues Organs* 148 96–109. 10.1159/000147529 8109201

[B83] Fritzsch B. Sarai P. Barbacid M. Silos-Santiago I. (1997). Mice with a targeted disruption of the neurotrophin receptor trkB lose their gustatory ganglion cells early but do develop taste buds. *Int. J. Dev. Neurosci.* 15 563–576. 10.1016/s0736-5748(96)00111-6 9263033

[B84] Gasparini S. Resch J. M. Gore A. M. Peltekian L. Geerling J. C. (2021). Pre-locus coeruleus neurons in rat and mouse. *Am. J. Physiol. Regul Integr. Comp. Physiol.* 320 R342–R361. 10.1152/ajpregu.00261.2020 33296280 PMC7988775

[B85] Glover J. C. (2020). “Development and evolution of vestibulo-ocular reflex circuitry,” in *The senses*, ed. Fritzsch B. (Amsterdam: Elsevier), 309–325. 10.1002/cne.902270408

[B86] Glover J. C. Elliott K. L. Erives A. Chizhikov V. V. Fritzsch B. (2018). Wilhelm His’ lasting insights into hindbrain and cranial ganglia development and evolution. *Dev. Biol.* 444 S14–S24. 10.1016/j.ydbio.2018.02.001 29447907 PMC6087689

[B87] Glover J. C. Renaud J. S. Rijli F. M. (2006). Retinoic acid and hindbrain patterning. *J. Neurobiol.* 66 705–725. 10.1002/neu.20272 16688767

[B88] Gogolla N. (2017). The insular cortex. *Curr. Biol.* 27 R580–R586. 10.1016/j.cub.2017.05.010 28633023

[B89] Goodman J. M. Bensmaia S. J. (2021). “The neural mechanisms of touch and proprioception at the somatosensory periphery,” in *The senses*, ed. Fritzsch B. (Amsterdam: Elsevier), 2–27. 10.1155/2020/3069639

[B90] Grabner C. P. Moser T. (2018). Individual synaptic vesicles mediate stimulated exocytosis from cochlear inner hair cells. *Proc. Natl. Acad. Sci. U.S.A.* 115 12811–12816. 10.1073/pnas.1811814115 30463957 PMC6294930

[B91] Grau-Perales A. Gallo M. (2020). The auditory context-dependent attenuation of taste neophobia depends on D1 dopamine receptor activity in mice. *Behav. Brain Res.* 391:112687. 10.1016/j.bbr.2020.112687 32437890

[B92] Grau-Perales A. B. Gámiz F. Gallo M. (2021). Effect of hippocampal 6-OHDA lesions on the contextual modulation of taste recognition memory. *Behav. Brain Res.* 409:113320. 10.1016/j.bbr.2021.113320 33901433

[B93] Gray P. (2013). Transcription factors define the neuroanatomical organization of the medullary reticular formation. *Front. Neuroanat.* 7:7. 10.3389/fnana.2013.00007 23717265 PMC3653110

[B94] Green B. G. Nachtigal D. (2012). Somatosensory factors in taste perception: Effects of active tasting and solution temperature. *Physiol. Behav.* 107 488–495. 10.1016/j.physbeh.2012.05.010 22609629 PMC3513519

[B95] Grothe B. (2021). “The auditory system function – An integrative perspective,” in *The senses*, ed. Fritzsch B. 10.1016/B978-0-12-805408-6.00009-9 (Amsterdam: Elsevier), 1–17.

[B96] Grothe B. Carr C. E. Casseday J. H. Fritzsch B. Köppl C. (2004). *The evolution of central pathways and their neural processing patterns, Evolution of the vertebrate auditory system.* 10.1007/978-1-4419-8957-4_10 Berlin: Springer, 289–359.

[B97] Gurung B. Fritzsch B. (2004). Time course of embryonic midbrain and thalamic auditory connection development in mice as revealed by carbocyanine dye tracing. *J. Comp. Neurol.* 479 309–327. 10.1002/cne.20328 15457503 PMC3901530

[B98] Hall J. M. Bell M. L. Finger T. E. (2003). Disruption of sonic hedgehog signaling alters growth and patterning of lingual taste papillae. *Dev. Biol.* 255 263–277. 10.1016/S0012-1606(02)00048-9 12648489

[B99] Han P. Müller L. Hummel T. (2021). Peri-threshold trigeminal stimulation with capsaicin increases taste sensitivity in humans. *Chemosens. Percept.* 15 1–7. 10.1007/s12078-021-09285-4

[B100] Hanamori T. Kunitake T. Kato K. Kannan H. (1998). Responses of neurons in the insular cortex to gustatory, visceral, and nociceptive stimuli in rats. *J. Neurophysiol.* 79 2535–2545. 10.1152/jn.1998.79.5.2535 9582226

[B101] Handler A. Ginty D. D. (2021). The mechanosensory neurons of touch and their mechanisms of activation. *Nat. Rev. Neurosci.* 22 521–537. 10.1038/s41583-021-00489-x 34312536 PMC8485761

[B102] Hans J. Broman J. van Domburg P. (2020). *The somatosensory system, clinical neuroanatomy.* 10.1007/978-3-030-41878-6_4 Berlin: Springer, 171–255.

[B103] Hauck P. Hecht H. (2019). Having a drink with Tchaikovsky: The crossmodal influence of background music on the taste of beverages. *Multisens. Res.* 32 1–24. 10.1163/22134808-20181321 31059491

[B104] Hawkes R. (2012). Pattern formation during development of the embryonic cerebellum. *Front. Neuroanat.* 6:10. 10.3389/fnana.2012.00010 22493569 PMC3318227

[B105] Herbert H. Moga M. M. Saper C. B. (1990). Connections of the parabrachial nucleus with the nucleus of the solitary tract and the medullary reticular formation in the rat. *J. Comp. Neurol.* 293 540–580. 10.1002/cne.902930404 1691748

[B106] Hernandez-Miranda L. R. Müller T. Birchmeier C. (2017). The dorsal spinal cord and hindbrain: From developmental mechanisms to functional circuits. *Dev. Biol.* 432 34–42. 10.1016/j.ydbio.2016.10.008 27742210

[B107] Hertzano R. Montcouquiol M. Rashi-Elkeles S. Elkon R. Yücel R. Frankel W. N. (2004). Transcription profiling of inner ears from Pou4f3 ddl/ddl identifies Gfi1 as a target of the Pou4f3 deafness gene. *Hum. Mol. Genet.* 13 2143–2153. 10.1093/hmg/ddh218 15254021

[B108] Hihara H. Kanetaka H. Kanno A. Shimada E. Koeda S. Kawashima R. (2020). Somatosensory evoked magnetic fields of periodontal mechanoreceptors. *Heliyon* 6:e03244. 10.1016/j.heliyon.2020.e03244 32021932 PMC6993012

[B109] Hill D. L. May O. L. (2007). “Development and plasticity of the gustatory portion of nucleus of the solitary tract,” in *The role of the nucleus of the solitary tract in gustatory processing*, ed. Bradley R. M. 10.1201/9781420005974.ch6 (Boca Raton, FL: CRC Press). 21204465

[B110] Hirsch D. Kohl A. Wang Y. Sela-Donenfeld D. (2021). Axonal projection patterns of the dorsal interneuron populations in the embryonic hindbrain. *Front. Neuroanat.* 15:793161. 10.3389/fnana.2021.793161 35002640 PMC8738170

[B111] Holcomb P. S. Hoffpauir B. K. Hoyson M. C. Jackson D. R. Deerinck T. J. Marrs G. S. (2013). Synaptic inputs compete during rapid formation of the calyx of Held: A new model system for neural development. *J. Neurosci.* 33 12954–12969. 10.1523/JNEUROSCI.1087-13.2013 23926251 PMC3735879

[B112] Holley M. Rhodes C. Kneebone A. Herde M. K. Fleming M. Steel K. P. (2010). Emx2 and early hair cell development in the mouse inner ear. *Dev. Biol.* 340 547–556. 10.1016/j.ydbio.2010.02.004 20152827 PMC2877772

[B113] Holt-Hansen K. (1968). Taste and pitch. *Percept. Mot. Skills* 27 59–68. 10.2466/pms.1968.27.1.59 5685718

[B114] Huang E. J. Liu W. Fritzsch B. Bianchi L. M. Reichardt L. F. Xiang M. (2001). Brn3a is a transcriptional regulator of soma size, target field innervation and axon pathfinding of inner ear sensory neurons. *Development* 128 2421–2432. 10.1242/dev.128.13.2421 11493560 PMC2710107

[B115] Huang E. J. Reichardt L. F. (2003). Trk receptors: Roles in neuronal signal transduction. *Annu. Rev. Biochem.* 72 609–642. 10.1146/annurev.biochem.72.121801.161629 12676795

[B116] Huang T. Krimm R. F. (2010). Developmental expression of Bdnf, Ntf4/5, and TrkB in the mouse peripheral taste system. *Dev. Dyn.* 239 2637–2646. 10.1002/dvdy.22412 21038447 PMC3058810

[B117] Hummel T. Iannilli E. Frasnelli J. Boyle J. Gerber J. (2009). Central processing of trigeminal activation in humans. *Ann. N. Y. Acad. Sci.* 1170 190–195. 10.1111/j.1749-6632.2009.03910.x 19686136

[B118] Iskusnykh I. Y. Steshina E. Y. Chizhikov V. V. (2016). Loss of Ptf1a leads to a widespread cell-fate misspecification in the brainstem, affecting the development of somatosensory and viscerosensory nuclei. *J. Neurosci.* 36 2691–2710. 10.1523/JNEUROSCI.2526-15.2016 26937009 PMC4879213

[B119] Iwasato T. Erzurumlu R. S. (2018). Development of tactile sensory circuits in the CNS. *Curr. Opin. Neurobiol.* 53 66–75. 10.1016/j.conb.2018.06.001 29908482 PMC6242754

[B120] Jahan I. Pan N. Kersigo J. Fritzsch B. (2010). Neurod1 suppresses hair cell differentiation in ear ganglia and regulates hair cell subtype development in the cochlea. *PLoS One* 5:e11661. 10.1371/journal.pone.0011661 20661473 PMC2908541

[B121] Jean P. Özçete Ö. D. Tarchini B. Moser T. (2019). Intrinsic planar polarity mechanisms influence the position-dependent regulation of synapse properties in inner hair cells. *Proc. Natl. Acad. Sci. U.S.A.* 116 9084–9093. 10.1073/pnas.1818358116 30975754 PMC6500111

[B122] Jiang T. Kindt K. Wu D. K. (2017). Transcription factor Emx2 controls stereociliary bundle orientation of sensory hair cells. *eLife* 6:e23661. 10.7554/eLife.23661 28266911 PMC5388538

[B123] Kageyama R. Shimojo H. Ohtsuka T. (2019). Dynamic control of neural stem cells by bHLH factors. *Neurosci. Res.* 138 12–18. 10.1016/j.neures.2018.09.005 30227160

[B124] Kandler K. Lee J. Pecka M. (2020). “2.28 – The superior olivary complex,” in *The senses: A comprehensive reference*, 2nd Edn, ed. Fritzsch B. (Oxford: Elsevier), 533–555. 10.1016/B978-0-12-805408-6.00021-X

[B125] Kandler K. Lee J. Pecka M. (2021). “The superior olivary complex,” in *The senses*, ed. Fritzsch B. (Amsterdam: Elsevier), 533–555.

[B126] Karthik S. Huan D. Delgado Y. Laing J. J. Peltekian L. Iversen G. (2022). Molecular ontology of the parabrachial nucleus. *J. Comp. Neurol.* 530 1658–1699. 10.1002/cne.25307 35134251 PMC9119955

[B127] Keast R. S. Breslin P. A. (2003). An overview of binary taste–taste interactions. *Food Qual. Prefer.* 14 111–124. 10.1016/S0950-3293(02)00110-6

[B128] Kersigo J. D’Angelo A. Gray B. D. Soukup G. A. Fritzsch B. (2011). The role of sensory organs and the forebrain for the development of the craniofacial shape as revealed by Foxg1-cre-mediated microRNA loss. *Genesis* 49 326–341. 10.1002/dvg.20714 21225654 PMC3079063

[B129] Kindt K. S. Akturk A. Jarysta A. Day M. Beirl A. Flonard M. (2021). EMX2-GPR156-Gαi reverses hair cell orientation in mechanosensory epithelia. *Nat. Commun.* 12:2861. 10.1038/s41467-021-22997-1 34001891 PMC8129141

[B130] King A. J. (2020). “Feedback systems: Descending pathways and adaptive coding in the auditory system,” in *The senses*, ed. Fritzsch B. (Amsterdam: Elsevier), 732–748. 10.7554/eLife.73289

[B131] Kinnamon S. C. Finger T. E. (2019). Recent advances in taste transduction and signaling. *F1000Res.* 8:F1000FacultyRev-2117. 10.12688/f1000research.21099.1 32185015 PMC7059786

[B132] Kobayashi T. Piao W. Takamura T. Kori H. Miyachi H. Kitano S. (2019). Enhanced lysosomal degradation maintains the quiescent state of neural stem cells. *Nat. Commun.* 10:5446. 10.1038/s41467-019-13203-4 31784517 PMC6884460

[B133] Kopecky B. Santi P. Johnson S. Schmitz H. Fritzsch B. (2011). Conditional deletion of N-Myc disrupts neurosensory and non-sensory development of the ear. *Dev. Dyn.* 240 1373–1390. 10.1002/dvdy.22620 21448975 PMC3092837

[B134] Kral A. Pallas S. L. (2011). “Development of the auditory cortex,” in *The auditory cortex*, eds Winer J. A. Schreiner C. E. (Berlin: Springer), 443–463. 10.1007/978-1-4419-0074-6_21

[B135] Kuypers H. G. Tuerk J. (1964). The distribution of cortical fibres within the nuclei cuneatus and gracilis in the cat. *J. Anat.* 98 143–162. 14154418 PMC1261271

[B136] Kwon H. G. Jang S. H. Lee M. Y. (2017). Effects of visual information regarding tactile stimulation on the somatosensory cortical activation: A functional MRI study. *Neural Regen. Res.* 12 1119–1123. 10.4103/1673-5374.211191 28852394 PMC5558491

[B137] Lai H. C. Seal R. P. Johnson J. E. (2016). Making sense out of spinal cord somatosensory development. *Development* 143 3434–3448. 10.1242/dev.139592 27702783 PMC5087618

[B138] Lazarov N. E. (2002). Comparative analysis of the chemical neuroanatomy of the mammalian trigeminal ganglion and mesencephalic trigeminal nucleus. *Prog. Neurobiol.* 66 19–59. 10.1016/s0301-0082(01)00021-1 11897404

[B139] Lee K. J. Dietrich P. Jessell T. M. (2000). Genetic ablation reveals that the roof plate is essential for dorsal interneuron specification. *Nature* 403 734–740. 10.1038/35001507 10693795

[B140] Lee S. Kruglikov I. Huang Z. J. Fishell G. Rudy B. (2013). A disinhibitory circuit mediates motor integration in the somatosensory cortex. *Nat. Neurosci.* 16 1662–1670. 10.1038/nn.3544 24097044 PMC4100076

[B141] Li J. Ali M. S. S. Lemon C. H. (2021). TRPV1-lineage somatosensory fibers communicate with taste neurons in the mouse parabrachial nucleus. *bioRxiv [Preprint]* 10.1523/JNEUROSCI.0927-21.2021 35027408 PMC8896561

[B142] Liao C.-C. Qi H.-X. Reed J. L. Kaas J. H. (2020). “The somatosensory system of primates,” in *The senses*, ed. Fritzsch B. 10.1016/B978-0-12-805408-6.00028-2 (Amsterdam: Elsevier), 180–197.

[B143] Lipovsek M. Ledderose J. Butts T. Lafont T. Kiecker C. Wizenmann A. (2017). The emergence of mesencephalic trigeminal neurons. *Neural Dev.* 12:11. 10.1186/s13064-017-0088-z 28637511 PMC5480199

[B144] Lipovsek M. Wingate R. J. (2018). Conserved and divergent development of brainstem vestibular and auditory nuclei. *eLife* 7:e40232. 10.7554/eLife.40232 30566077 PMC6317910

[B145] Liu F. Thirumangalathu S. Gallant N. M. Yang S. H. Stoick-Cooper C. L. Reddy S. T. (2007). Wnt-beta-catenin signaling initiates taste papilla development. *Nat. Genet.* 39 106–112. 10.1038/ng1932 17128274

[B146] Lorenzen S. M. Duggan A. Osipovich A. B. Magnuson M. A. García-Añoveros J. (2015). Insm1 promotes neurogenic proliferation in delaminated otic progenitors. *Mech. Dev.* 138(Pt 3), 233–245. 10.1016/j.mod.2015.11.001 26545349 PMC4679445

[B147] Lorenzo P. M. D. (2021). Neural coding of food is a multisensory, sensorimotor function. *Nutrients* 13:398. 10.3390/nu13020398 33513918 PMC7911409

[B148] Loutit A. J. Vickery R. M. Potas J. R. (2021). Functional organization and connectivity of the dorsal column nuclei complex reveals a sensorimotor integration and distribution hub. *J. Comp. Neurol.* 529 187–220. 10.1002/cne.24942 32374027

[B149] Lowenstein E. D. Cui K. Hernandez-Miranda L. R. (2022). Regulation of early cerebellar development. *FEBS J.* [Epub ahead of print]. 10.1111/febs.16426 35262281

[B150] Lunde A. Okaty B. W. Dymecki S. M. Glover J. C. (2019). Molecular profiling defines evolutionarily conserved transcription factor signatures of major vestibulospinal neuron groups. *Eneuro* 6:ENEURO.0475-18.2019. 10.1523/ENEURO.0475-18.2019 30899776 PMC6426439

[B151] Lundy R. F. Jr. Norgren R. (2015). “Gustatory system,” in *The rat nervous system*, ed. Paxinos G. 10.1016/B978-0-12-374245-2.00026-7 (Amsterdam: Elsevier), 733–760.

[B152] Ma Q. Anderson D. J. Fritzsch B. (2000). Neurogenin 1 null mutant ears develop fewer, morphologically normal hair cells in smaller sensory epithelia devoid of innervation. *J. Assoc. Res. Otolaryngol.* 1 129–143. 10.1007/s101620010017 11545141 PMC2504536

[B153] Ma Q. Chen Z. del Barco Barrantes I. De La Pompa J. L. Anderson D. J. (1998). neurogenin1 is essential for the determination of neuronal precursors for proximal cranial sensory ganglia. *Neuron* 20 469–482. 10.1016/s0896-6273(00)80988-5 9539122

[B154] Ma Q. Fode C. Guillemot F. Anderson D. J. (1999). Neurogenin1 and neurogenin2 control two distinct waves of neurogenesis in developing dorsal root ganglia. *Genes Dev.* 13 1717–1728. 10.1101/gad.13.13.1717 10398684 PMC316844

[B155] Macova I. Pysanenko K. Chumak T. Dvorakova M. Bohuslavova R. Syka J. (2019). Neurod1 is essential for the primary tonotopic organization and related auditory information processing in the midbrain. *J. Neurosci.* 39 984–1004. 10.1523/JNEUROSCI.2557-18.2018 30541910 PMC6363931

[B156] Majka P. Rosa M. G. Bai S. Chan J. M. Huo B.-X. Jermakow N. (2019). Unidirectional monosynaptic connections from auditory areas to the primary visual cortex in the marmoset monkey. *Brain Struct. Funct.* 224 111–131. 10.1007/s00429-018-1764-4 30288557 PMC6373361

[B157] Maklad A. Fritzsch B. (1999). Incomplete segregation of endorgan-specific vestibular ganglion cells in mice and rats. *J. Vestib. Res.* 9 387–399. 10.3233/VES-1999-9601 10639024

[B158] Maklad A. Fritzsch B. (2003). Partial segregation of posterior crista and saccular fibers to the nodulus and uvula of the cerebellum in mice, and its development. *Brain Res. Dev. Brain Res.* 140 223–236. 10.1016/s0165-3806(02)00609-0 12586428

[B159] Malmierca M. S. (2015). “Auditory system,” in *The rat nervous system*, ed. Paxinos G. (Cambridge, MA: Academic Press), 865–946.

[B160] Malone B. J. Hasenstaub A. R. Schreiner C. E. (2021). “Primary auditory cortex II. Some functional considerations,” in *The senses*, ed. Fritzsch B. 10.1016/B978-0-12-809324-5.24268-5 (Amsterdam: Elsevier), 657–680.

[B161] Mao C.-A. Cho J.-H. Wang J. Gao Z. Pan P. Tsai W.-W. (2013). Reprogramming amacrine and photoreceptor progenitors into retinal ganglion cells by replacing Neurod1 with Atoh7. *Development* 140 541–551. 10.1242/dev.085886 23293286 PMC3561791

[B162] Maricich S. M. Wellnitz S. A. Nelson A. M. Lesniak D. R. Gerling G. J. Lumpkin E. A. (2009). Merkel cells are essential for light-touch responses. *Science* 324 1580–1582. 10.1126/science.1172890 19541997 PMC2743005

[B163] Marrs G. S. Morgan W. J. Howell D. M. Spirou G. A. Mathers P. H. (2013). Embryonic origins of the mouse superior olivary complex. *Dev. Neurobiol.* 73 384–398. 10.1002/dneu.22069 23303740 PMC4217651

[B164] Marrs G. S. Spirou G. A. (2012). Embryonic assembly of auditory circuits: Spiral ganglion and brainstem. *J. Physiol.* 590 2391–2408. 10.1113/jphysiol.2011.226886 22371481 PMC3424760

[B165] Marzban H. Rahimi-Balaei M. Hawkes R. (2019). Early trigeminal ganglion afferents enter the cerebellum before the Purkinje cells are born and target the nuclear transitory zone. *Brain Struct. Funct.* 224, 2421–2436. 31256239 10.1007/s00429-019-01916-7

[B166] Matei V. Pauley S. Kaing S. Rowitch D. Beisel K. Morris K. (2005). Smaller inner ear sensory epithelia in Neurog1 null mice are related to earlier hair cell cycle exit. *Dev. Dyn.* 234 633–650. 10.1002/dvdy.20551 16145671 PMC1343505

[B167] Matsuo S. Ichikawa H. Silos-Santiago I. Arends J. Henderson T. Kiyomiya K. (1999). Proprioceptive afferents survive in the masseter muscle of trkC knockout mice. *Neuroscience* 95 209–216. 10.1016/s0306-4522(99)00424-8 10619477

[B168] Meehan T. P. Bressler S. L. Tang W. Astafiev S. V. Sylvester C. M. Shulman G. L. (2017). Top-down cortical interactions in visuospatial attention. *Brain Struct. Funct.* 222 3127–3145. 10.1007/s00429-017-1390-6 28321551 PMC5607080

[B169] Meltzer S. Santiago C. Sharma N. Ginty D. D. (2021). The cellular and molecular basis of somatosensory neuron development. *Neuron* 109 3736–3757. 10.1016/j.neuron.2021.09.004 34592169 PMC8639614

[B170] Mishima Y. Lindgren A. G. Chizhikov V. V. Johnson R. L. Millen K. J. (2009). Overlapping function of Lmx1a and Lmx1b in anterior hindbrain roof plate formation and cerebellar growth. *J. Neurosci.* 29 11377–11384. 10.1523/JNEUROSCI.0969-09.2009 19741143 PMC2765661

[B171] Mizoguchi N. Muramoto K. Kobayashi M. (2020). Olfactory signals from the main olfactory bulb converge with taste information from the chorda tympani nerve in the agranular insular cortex of rats. *Pflügers Arch.* 472 721–732. 10.1007/s00424-020-02399-w 32458087

[B172] Montcouquiol M. Rachel R. A. Lanford P. J. Copeland N. G. Jenkins N. A. Kelley M. W. (2003). Identification of Vangl2 and Scrb1 as planar polarity genes in mammals. *Nature* 423 173–177. 10.1038/nature01618 12724779

[B173] Moro S. S. Harris L. R. (2018). Vestibular–somatosensory interactions affect the perceived timing of tactile stimuli. *Exp. Brain Res.* 236 2877–2885. 10.1007/s00221-018-5346-8 30062442

[B174] Moser T. (2020). “Presynaptic physiology of cochlear inner hair cells,” in *The senses*, ed. Fritzsch B. (Amsterdam: Elsevier), 441–467. 10.1016/B978-0-12-809324-5.24185-0

[B175] Murray M. M. Molholm S. Michel C. M. Heslenfeld D. J. Ritter W. Javitt D. C. (2005). Grabbing your ear: Rapid auditory–somatosensory multisensory interactions in low-level sensory cortices are not constrained by stimulus alignment. *Cereb. Cortex* 15 963–974. 10.1093/cercor/bhh197 15537674

[B176] Nakano Y. Wiechert S. Fritzsch B. Bánfi B. (2020). Inhibition of a transcriptional repressor rescues hearing in a splicing factor–deficient mouse. *Life Sci. Alliance* 3:e202000841. 10.26508/lsa.202000841 33087486 PMC7652395

[B177] Nichols D. H. Bruce L. L. (2006). Migratory routes and fates of cells transcribing the Wnt-1 gene in the murine hindbrain. *Dev. Dyn.* 235 285–300. 10.1002/dvdy.20611 16273520

[B178] Nosrat I. V. Margolskee R. F. Nosrat C. A. (2012). Targeted taste cell-specific overexpression of brain-derived neurotrophic factor in adult taste buds elevates phosphorylated TrkB protein levels in taste cells, increases taste bud size, and promotes gustatory innervation. *J. Biol. Chem.* 287 16791–16800. 10.1074/jbc.M111.328476 22442142 PMC3351349

[B179] Obana E. A. Zhou Q. Furmanski O. Doughty M. L. (2018). Conditional deletion of Neurog1 in the cerebellum of postnatal mice delays inhibitory interneuron maturation. *J. Neurosci. Res.* 96 1560–1575. 10.1002/jnr.24247 29665106

[B180] Oertel D. Cao X.-J. (2020). “2.27 – The ventral cochlear nucleus,” in *The senses: A comprehensive reference*, 2nd Edn, ed. Fritzsch B. 10.1016/B978-0-12-809324-5.23880-7 (Oxford: Elsevier), 517–532.

[B181] Ohmoto M. Kitamoto S. Hirota J. (2020). Expression of Eya1 in mouse taste buds. *Cell Tissue Res.* 383 979–986. 10.1007/s00441-020-03311-9 33242174

[B182] Ohmoto M. Ren W. Nishiguchi Y. Hirota J. Jiang P. Matsumoto I. (2017). Genetic lineage tracing in taste tissues using Sox2-CreERT2 strain. *Chem. Senses* 42 547–552. 10.1093/chemse/bjx032 28595328 PMC6075561

[B183] Okubo T. Pevny L. H. Hogan B. L. (2006). Sox2 is required for development of taste bud sensory cells. *Genes Dev.* 20 2654–2659. 17015430 10.1101/gad.1457106 PMC1578692

[B184] O’Neill P. Mak S.-S. Fritzsch B. Ladher R. K. Baker C. V. (2012). The amniote paratympanic organ develops from a previously undiscovered sensory placode. *Nat. Commun.* 3:1041. 10.1038/ncomms2036 22948823 PMC3518548

[B185] Ossenkopp K.-P. Parker L. A. Limebeer C. L. Burton P. Fudge M. A. Cross-Mellor S. K. (2003). Vestibular lesions selectively abolish body rotation-induced, but not lithium-induced, conditioned taste aversions (oral rejection responses) in rats. *Behav. Neurosci.* 117 105–112. 10.1037/0735-7044.117.1.105 12619913

[B186] O’Sullivan A. E. Crosse M. J. Di Liberto G. M. de Cheveigné A. Lalor E. C. (2021). Neurophysiological indices of audiovisual speech processing reveal a hierarchy of multisensory integration effects. *J. Neurosci.* 41 4991–5003. 10.1523/JNEUROSCI.0906-20.2021 33824190 PMC8197638

[B187] Pallas S. L. (2001). Intrinsic and extrinsic factors that shape neocortical specification. *Trends Neurosci.* 24 417–423. 10.1016/s0166-2236(00)01853-1 11410273

[B188] Pan B. Géléoc G. S. Asai Y. Horwitz G. C. Kurima K. Ishikawa K. (2013). TMC1 and TMC2 are components of the mechanotransduction channel in hair cells of the mammalian inner ear. *Neuron* 79 504–515. 10.1016/j.neuron.2013.06.019 23871232 PMC3827726

[B189] Pauley S. Lai E. Fritzsch B. (2006). Foxg1 is required for morphogenesis and histogenesis of the mammalian inner ear. *Dev. Dyn.* 235 2470–2482. 10.1002/dvdy.20839 16691564 PMC3901532

[B190] Pei Y.-C. Hsiao S. S. Craig J. C. Bensmaia S. J. (2011). Neural mechanisms of tactile motion integration in somatosensory cortex. *Neuron* 69 536–547. 10.1016/j.neuron.2010.12.033 21315263 PMC3052381

[B191] Perea-Martinez I. Nagai T. Chaudhari N. (2013). Functional cell types in taste buds have distinct longevities. *PLoS One* 8:e53399. 10.1371/journal.pone.0053399 23320081 PMC3540047

[B192] Peterka R. J. Loughlin P. J. (2004). Dynamic regulation of sensorimotor integration in human postural control. *J. Neurophysiol.* 91 410–423. 10.1152/jn.00516.2003 13679407

[B193] Petersen C. I. Jheon A. H. Mostowfi P. Charles C. Ching S. Thirumangalathu S. (2011). FGF signaling regulates the number of posterior taste papillae by controlling progenitor field size. *PLoS Genet.* 7:e1002098. 10.1371/journal.pgen.1002098 21655085 PMC3107195

[B194] Pfeiffer C. Noel J. P. Serino A. Blanke O. (2018). Vestibular modulation of peripersonal space boundaries. *Eur. J. Neurosci.* 47 800–811. 10.1111/ejn.13872 29461657

[B195] Pheasant R. J. Fisher M. N. Watts G. R. Whitaker D. J. Horoshenkov K. V. (2010). The importance of auditory-visual interaction in the construction of ‘tranquil space’. *J. Environ. Psychol.* 30 501–509. 10.1016/j.jenvp.2010.03.006

[B196] Phillips J. O. Ling L. Nowack A. Rebollar B. Rubinstein J. T. (2020). Interactions between auditory and vestibular modalities during stimulation with a combined vestibular and cochlear prosthesis. *Audiol. Neurotol.* 25 96–108. 10.1159/000503846 31968338 PMC7050683

[B197] Phillips-Silver J. Trainor L. J. (2008). Vestibular influence on auditory metrical interpretation. *Brain Cogn.* 67 94–102. 10.1016/j.bandc.2007.11.007 18234407

[B198] Phillips-Silver J. VanMeter J. W. Rauschecker J. P. (2020). Auditory-vestibulomotor temporal processing and crossmodal plasticity for musical rhythm in the early blind. *bioRxiv [Preprint]* 10.1101/2020.03.23.987727

[B199] Plass J. Brang D. Suzuki S. Grabowecky M. (2020). Vision perceptually restores auditory spectral dynamics in speech. *Proc. Natl. Acad. Sci. U.S.A.* 117 16920–16927. 10.1073/pnas.2002887117 32632010 PMC7382243

[B200] Pop I. V. Espinosa F. Blevins C. J. Okafor P. C. Ogujiofor O. W. Goyal M. (2021). Structure of long-range direct and indirect spinocerebellar pathways as well as local spinal circuits mediating proprioception. *J. Neurosci.* 42 581–600. 10.1523/JNEUROSCI.2157-20.2021 34857649 PMC8805613

[B201] Pritchard T. C. Macaluso D. A. Eslinger P. J. (1999). Taste perception in patients with insular cortex lesions. *Behav. Neurosci.* 113 663–671. 10.1037/0735-7044.113.4.663 10495075

[B202] Qi H.-X. Liao C.-C. Reed J. L. Kaas J. H. (2020). “Cortical and subcortical plasticity after sensory loss in the somatosensory system of primates,” in *The senses*, ed. Fritzsch B. 10.1016/B978-0-12-809324-5.24228-4 (Amsterdam: Elsevier), 399–418.

[B203] Qian Y. Fritzsch B. Shirasawa S. Chen C.-L. Choi Y. Ma Q. (2001). Formation of brainstem (nor) adrenergic centers and first-order relay visceral sensory neurons is dependent on homeodomain protein Rnx/Tlx3. *Genes Dev.* 15 2533–2545. 10.1101/gad.921501 11581159 PMC312792

[B204] Rauschecker J. P. (2021). “The auditory cortex of primates including man with reference to speech,” in *The senses*, ed. Fritzsch B. 10.1016/B978-0-12-805408-6.00029-4 (Amsterdam: Elsevier), 791–811.

[B205] Rauschecker J. P. Scott S. K. (2009). Maps and streams in the auditory cortex: Nonhuman primates illuminate human speech processing. *Nat. Neurosci.* 12 718–724. 10.1038/nn.2331 19471271 PMC2846110

[B206] Razak K. A. Zumsteg T. Fuzessery Z. M. (2009). Development of auditory thalamocortical connections in the pallid bat, *Antrozous pallidus*. *J. Comp. Neurol.* 515 231–242. 10.1002/cne.22050 19412955 PMC2688833

[B207] Reiprich S. Wegner M. (2015). From CNS stem cells to neurons and glia: Sox for everyone. *Cell Tissue Res.* 359 111–124. 10.1007/s00441-014-1909-6 24894327

[B208] Rhyu M.-R. Kim Y. Lyall V. (2021). Interactions between chemesthesis and taste: Role of TRPA1 and TRPV1. *Int. J. Mol. Sci.* 22:3360. 10.3390/ijms22073360 33806052 PMC8038011

[B209] Riccomagno M. M. Martinu L. Mulheisen M. Wu D. K. Epstein D. J. (2002). Specification of the mammalian cochlea is dependent on sonic hedgehog. *Genes Dev.* 16 2365–2378. 10.1101/gad.1013302 12231626 PMC187441

[B210] Roper S. (2020). “Microphysiology of taste buds,” in *The senses*, ed. Fritzsch B. (Amsterdam: Elsevier), 187–210. 10.1523/JNEUROSCI.12-04-01127.1992

[B211] Roper S. D. (2021). Encoding taste: From receptors to perception. *Handb. Exp. Pharmacol.* 275 53–90. 10.1007/164_2021_559 PMC9744258 34796381

[B212] Roper S. D. Chaudhari N. (2017). Taste buds: Cells, signals and synapses. *Nat. Rev. Neurosci.* 18 485–497. 10.1038/nrn.2017.68 28655883 PMC5958546

[B213] Roper S. D. Krimm R. F. Fritzsch B. (2022). in *Taste buds explained: From taste sensing to taste processing in the forebrain*, eds Fritzsch B. Elliott K. L. 10.1201/9781003092810-5 (Boca Raton, FL: Wiley), 111–134.

[B214] Rose M. F. Ahmad K. A. Thaller C. Zoghbi H. Y. (2009). Excitatory neurons of the proprioceptive, interoceptive, and arousal hindbrain networks share a developmental requirement for Math1. *Proc. Natl. Acad. Sci. U.S.A.* 106 22462–22467. 10.1073/pnas.0911579106 20080794 PMC2799716

[B215] Saper C. Loewy A. (1980). Efferent connections of the parabrachial nucleus in the rat. *Brain Res.* 197 291–317. 10.1016/0006-8993(80)91117-8 7407557

[B216] Schier L. A. Spector A. C. (2019). The functional and neurobiological properties of bad taste. *Physiol. Rev.* 99 605–663. 10.1152/physrev.00044.2017 30475657 PMC6442928

[B217] Shaikh A. G. Zee D. S. Taube J. Kheradmand A. (2020). “Visual–vestibular interactions,” in *Multisensory perception*, 10.1016/B978-0-12-812492-5.00009-7 (Amsterdam: Elsevier), 201–219.

[B218] Shepherd G. M. (2006). Smell images and the flavour system in the human brain. *Nature* 444 316–321. 10.1038/nature05405 17108956

[B219] Shibata S. B. Ranum P. T. Moteki H. Pan B. Goodwin A. T. Goodman S. S. (2016). RNA interference prevents autosomal-dominant hearing loss. *Am. J. Hum. Genet.* 98 1101–1113. 10.1016/j.ajhg.2016.03.028 27236922 PMC4908151

[B220] Simmons D. Duncan J. de Caprona D. C. Fritzsch B. (2011). “Development of the inner ear efferent system,” in *Auditory and vestibular efferents*, 10.1007/978-1-4419-7070-1_7 (New York, NY: Springer), 187–216.

[B221] Smith A. C. Fleenor S. J. Begbie J. (2015). Changes in gene expression and cell shape characterise stages of epibranchial placode-derived neuron maturation in the chick. *J. Anat.* 227 89–102. 10.1111/joa.12333 26076761 PMC4475362

[B222] Spence C. (2011). Crossmodal correspondences: A tutorial review. *Atten. Percept. Psychophys.* 73 971–995. 10.3758/s13414-010-0073-7 21264748

[B223] Spence C. (2022). Multisensory contributions to affective touch. *Curr. Opin. Behav. Sci.* 43 40–45. 10.1016/j.cobeha.2021.08.003

[B224] Spence C. Ngo M. K. (2012). Assessing the shape symbolism of the taste, flavour, and texture of foods and beverages. *Flavour* 1 1–13. 10.1186/2044-7248-1-12

[B225] Spence C. Soto-Faraco S. (2010). Auditory perception: Interactions with vision. *Oxford Handb. Audit. Sci. Hear.* 3 271–296. 10.1093/oxfordhb/9780199233557.013.0012

[B226] Spence C. Wang Q. J. Reinoso-Carvalho F. Keller S. (2021). Commercializing sonic seasoning in multisensory offline experiential events and online tasting experiences. *Front. Psychol.* 12:740354. 10.3389/fpsyg.2021.740354 34659056 PMC8514999

[B227] Staszko S. Boughter J. (2020). “Taste pathways, representation and processing in the brain,” in *The senses*, ed. Fritzsch B. 10.1016/B978-0-12-809324-5.23891-1 (Amsterdam: Elsevier), 280–297.

[B228] Staszko S. M. Boughter J. D. Jr. Fletcher M. L. (2020). Taste coding strategies in insular cortex. *Exp. Biol. Med.* 245 448–455. 10.1177/1535370220909096 32106700 PMC7082883

[B229] Steiner L. Federspiel A. Slavova N. Wiest R. Grunt S. Steinlin M. (2020). Functional topography of the thalamo-cortical system during development and its relation to cognition. *Neuroimage* 223:117361. 10.1016/j.neuroimage.2020.117361 32919055

[B230] Stoner Z. A. Ketchum E. M. Sheltz-Kempf S. Blinkiewicz P. V. Elliott K. L. Duncan J. S. (2021). Fzd3 expression within inner ear afferent neurons is necessary for central pathfinding. *Front. Neurosci.* 15:779871. 10.3389/fnins.2021.779871 35153658 PMC8828977

[B231] Straka H. Fritzsch B. Glover J. C. (2014). Connecting ears to eye muscles: Evolution of a ‘simple’ reflex arc. *Brain Behav. Evol.* 83 162–175. 10.1159/000357833 24776996

[B232] Striedter G. F. Northcutt R. G. (2019). *Brains through time: A natural history of vertebrates.* 10.1093/oso/9780195125689.001.0001 Oxford: Oxford University Press.

[B233] Suer M. (2021). “Anatomy of the trigeminal nerve,” in *Trigeminal nerve pain: A guide to clinical management*, ed. Abd-Elsayed A. (Cham: Springer International Publishing), 5–16.

[B234] Sugiyama S. Takeuchi N. Inui K. Nishihara M. Shioiri T. (2018). Effect of acceleration of auditory inputs on the primary somatosensory cortex in humans. *Sci. Rep.* 8:12883. 30150686 10.1038/s41598-018-31319-3 PMC6110726

[B235] Suryanarayana S. M. Pérez-Fernández J. Robertson B. Grillner S. (2020). The evolutionary origin of visual and somatosensory representation in the vertebrate pallium. *Nat. Ecol. Evol.* 4 639–651. 32203472 10.1038/s41559-020-1137-2

[B236] Suryanarayana S. M. Pérez-Fernández J. Robertson B. Grillner S. (2021). Olfaction in lamprey pallium revisited—Dual projections of mitral and tufted cells. *Cell Rep.* 34:108596. 10.1016/j.celrep.2020.108596 33406414

[B237] Syka J. (2020). *Age-related changes in the auditory brainstem and inferior colliculus, aging and hearing.* Berlin: Springer, 67–96.

[B238] Taube J. S. Yoder R. M. (2021). “The impact of vestibular signals on cells responsible for orientation and navigation,” in *The senses*, ed. Fritzsch B. (Amsterdam: Elsevier), 496–511.

[B239] Teng B. Wilson C. E. Tu Y.-H. Joshi N. R. Kinnamon S. C. Liman E. R. (2019). Cellular and neural responses to sour stimuli require the proton channel Otop1. *Curr. Biol.* 29 3647–3656.e5. 10.1016/j.cub.2019.08.077 31543453 PMC7299528

[B240] Ter-Avetisyan G. Dumoulin A. Herrel A. Schmidt H. Strump J. Afzal S. (2018). Loss of axon bifurcation in mesencephalic trigeminal neurons impairs the maximal biting force in Npr2-deficient mice. *Front. Cell. Neurosci.* 12:153. 10.3389/fncel.2018.00153 29962937 PMC6013911

[B241] Tichko P. Kim J. C. Large E. W. (2021). Bouncing the network: A dynamical systems model of auditory–vestibular interactions underlying infants’ perception of musical rhythm. *Dev. Sci.* 24:e13103. 10.1111/desc.13103 33570778

[B242] Tosches M. A. Laurent G. (2019). Evolution of neuronal identity in the cerebral cortex. *Curr. Opin. Neurobiol.* 56 199–208. 31103814 10.1016/j.conb.2019.04.009

[B243] Trudeau-Fisette P. Ito T. Ménard L. (2019). Auditory and somatosensory interaction in speech perception in children and adults. *Front. Hum. Neurosci.* 13:344. 10.3389/fnhum.2019.00344 31636554 PMC6788346

[B244] Uddin L. Q. Nomi J. S. Hébert-Seropian B. Ghaziri J. Boucher O. (2017). Structure and function of the human insula. *J. Clin. Neurophysiol.* 34 300–306. 28644199 10.1097/WNP.0000000000000377 PMC6032992

[B245] Urness L. D. Bleyl S. B. Wright T. J. Moon A. M. Mansour S. L. (2011). Redundant and dosage sensitive requirements for Fgf3 and Fgf10 in cardiovascular development. *Dev. Biol.* 356 383–397. 10.1016/j.ydbio.2011.05.671 21664901 PMC3143275

[B246] Vendrell-Llopis N. Yaksi E. (2015). Evolutionary conserved brainstem circuits encode category, concentration and mixtures of taste. *Sci. Rep.* 5:17825. 10.1038/srep17825 26639368 PMC4671064

[B247] von Bartheld C. S. Fritzsch B. (2006). Comparative analysis of neurotrophin receptors and ligands in vertebrate neurons: Tools for evolutionary stability or changes in neural circuits? *Brain Behav. Evol.* 68 157–172. 16912469 10.1159/000094085

[B248] Wang Q. J. Keller S. Spence C. (2021). Metacognition and crossmodal correspondences between auditory attributes and saltiness in a large sample study. *Multisens. Res.* 34 785–805. 10.1163/22134808-bja10055 34375946

[B249] Wang V. Y. Rose M. F. Zoghbi H. Y. (2005). Math1 expression redefines the rhombic lip derivatives and reveals novel lineages within the brainstem and cerebellum. *Neuron* 48 31–43. 10.1016/j.neuron.2005.08.024 16202707

[B250] Watson C. Mitchelle A. Puelles L. (2017a). 2.02 A new mammalian brain ontology based on developmental gene expression. *Evol. Nervous Syst.*

[B251] Watson C. Shimogori T. Puelles L. (2017b). Mouse Fgf8-Cre-LacZ lineage analysis defines the territory of the postnatal mammalian isthmus. *J. Comp. Neurol.* 525 2782–2799. 10.1002/cne.24242 28510270

[B252] Wistehube T. Rullmann M. Wiacek C. Braun P. Pleger B. (2018). Fat perception in the human frontal operculum, insular and somatosensory cortex. *Sci. Rep.* 8:11825. 10.1038/s41598-018-30366-0 30087417 PMC6081453

[B253] Wiwatpanit T. Lorenzen S. M. Cantú J. A. Foo C. Z. Hogan A. K. Márquez F. (2018). Trans-differentiation of outer hair cells into inner hair cells in the absence of INSM1. *Nature* 563 691–695. 30305733 10.1038/s41586-018-0570-8 PMC6279423

[B254] Wooding S. Ramirez V. (2020). “Taste genetics,” in *The senses*, ed. Fritzsch B. (Amsterdam: Elsevier), 264–279.

[B255] Wullimann M. F. Grothe B. (2013). “The central nervous organization of the lateral line system,” in *The lateral line system*, eds Coombs S. Bleckmann H. Fay R. Popper A. (New York, NY: Springer), 195–251.

[B256] Xiang M. Maklad A. Pirvola U. Fritzsch B. (2003). Brn3c null mutant mice show long-term, incomplete retention of some afferent inner ear innervation. *BMC Neurosci.* 4:2. 10.1186/1471-2202-4-2 12585968 PMC149366

[B257] Xu J. Li J. Zhang T. Jiang H. Ramakrishnan A. Fritzsch B. (2021). Chromatin remodelers and lineage-specific factors interact to target enhancers to establish proneurosensory fate within otic ectoderm. *Proc. Natl. Acad. Sci. U.S.A.* 118:e2025196118. 10.1073/pnas.2025196118 33723076 PMC8000026

[B258] Yamada M. Seto Y. Taya S. Owa T. Inoue Y. U. Inoue T. (2014). Specification of spatial identities of cerebellar neuron progenitors by ptf1a and atoh1 for proper production of GABAergic and glutamatergic neurons. *J. Neurosci.* 34 4786–4800. 10.1523/JNEUROSCI.2722-13.2014 24695699 PMC6802724

[B259] Yang R. Dzowo Y. K. Wilson C. E. Russell R. L. Kidd G. J. Salcedo E. (2020). Three-dimensional reconstructions of mouse circumvallate taste buds using serial blockface scanning electron microscopy: I. Cell types and the apical region of the taste bud. *J. Comp. Neurol.* 528 756–771. 10.1002/cne.24779 31587284 PMC7041425

[B260] Zampini M. Spence C. (2012). “Assessing the role of visual and auditory cues in multisensory perception of flavor,” in *The neural bases of multisensory processes*, eds Murray M. M. Wallace M. T. (Boca Raton, FL: CRC Press). 22593877

[B261] Zhang M. Kwon S. E. Ben-Johny M. O’Connor D. H. Issa J. B. (2020). Spectral hallmark of auditory-tactile interactions in the mouse somatosensory cortex. *Commun. Biol.* 3:64. 10.1038/s42003-020-0788-5 32047263 PMC7012892

[B262] Zhang Y. Lu W.-J. Bulkley D. P. Liang J. Ralko A. Han S. (2020). Hedgehog pathway activation through nanobody-mediated conformational blockade of the Patched sterol conduit. *Proc. Natl. Acad. Sci. U.S.A.* 117 28838–28846. 10.1073/pnas.2011560117 33139559 PMC7682405

[B263] Zhang T. Xu J. Xu P. X. (2021). Eya2 expression during mouse embryonic development revealed by Eya2(lacZ) knockin reporter and homozygous mice show mild hearing loss. *Dev. Dyn.* 250 1450–1462. 10.1002/dvdy.326 33715274 PMC8438093

[B264] Zhao J. Xu W. Ye L. (2018). Effects of auditory-visual combinations on perceived restorative potential of urban green space. *Appl. Acoust.* 141 169–177.

[B265] Zhou G. Lane G. Noto T. Arabkheradmand G. Gottfried J. A. Schuele S. U. (2019). Human olfactory-auditory integration requires phase synchrony between sensory cortices. *Nat. Commun.* 10:1168. 10.1038/s41467-019-09091-3 30858379 PMC6411726

[B266] Zhu Z. Disbrow E. A. Zumer J. M. McGonigle D. J. Nagarajan S. S. (2007). Spatiotemporal integration of tactile information in human somatosensory cortex. *BMC Neurosci.* 8:21. 10.1186/1471-2202-8-21 17359544 PMC1838913

[B267] Zilles K. Palomero-Gallagher N. (2021). “The architecture of somatosensory cortex,” in *The senses*, ed. Fritzsch B. 10.1016/B978-0-12-809324-5.24128-X (Amsterdam: Elsevier), 225–260.

[B268] Zou D. Silvius D. Fritzsch B. Xu P.-X. (2004). Eya1 and Six1 are essential for early steps of sensory neurogenesis in mammalian cranial placodes. *Development* 131 5561–5572. 10.1242/dev.01437 15496442 PMC3882150

